# Long-term outcomes after kidney transplant failure and variables related to risk of death and probability of retransplant: Results from a single-center cohort study in Brazil

**DOI:** 10.1371/journal.pone.0245628

**Published:** 2021-01-20

**Authors:** Lúcio R. Requião-Moura, Cássio R. Moreira Albino, Paula Rebello Bicalho, Érika de Arruda Ferraz, Luciana Mello de Mello Barros Pires, Maurício Fregonesi Rodrigues da Silva, Alvaro Pacheco-Silva

**Affiliations:** 1 Renal Transplant Unit, Hospital Israelita Albert Einstein, São Paulo, Brazil; 2 Nephrology Division, Federal University of São Paulo, São Paulo, Brazil; Medical University of Gdansk, POLAND

## Abstract

**Background:**

Returning to dialysis after kidney graft loss (GL) is associated with a high risk of mortality, mainly in the first 3–6 months. The follow-up of patients with GL should be extended to better understand crude patient outcomes, mainly in emerging countries, where the transplantation activity has increased.

**Methods:**

This is a historical single-center cohort study conducted in an emerging country (Brazil) that included 115 transplant patients with kidney allograft failure who were followed for 44.1 (21.4; 72.6) months after GL. The outcomes were death or retransplantation after GL calculated by Kaplan-Meier and log-rank tests. Proportional hazard ratios for death and retransplantation were assessed by Cox regression.

**Results:**

The 5-year probability of retransplantation was 38.7% (95% CI: 26.1%-51.2%) and that of death was 37.7% (95% CI: 24.9%-50.5%); OR = 1.03 (95% CI: 0.71–1.70) and P = 0.66. The likelihood of retransplantation was higher in patients who resumed dialysis with higher levels of hemoglobin (HR = 1.22; 95% CI = 1.04–1.43; P = 0.01) and lower in blood type O patients (HR = 0.48; 95% CI = 0.25–0.93; P = 0.03), which was associated with a lower frequency of retransplantation with a subsequent living-donor kidney. On the other hand, the risk of death was significantly associated with Charlson comorbidity index (HR for each point = 1.37; 95% CI 1.19–1.50; P<0.001), and residual eGFR at the time when patients had resumed to dialysis (HR for each mL = 1.14; 95% CI = 1.05–1.25; P = 0.002). The trend toward a lower risk of death when patients had resumed to dialysis using AV fistula access was observed (HR = 0.50; 95% CI 0.25–1.02; P = 0.06), while a higher risk seems to be associated with the number of previous engraftment (HR = 2.01; 95% CI 0.99–4.07; P = 0.05).

**Conclusions:**

The 5-year probability of retransplantation was not less than that of death. Variables related to the probability of retransplantation were hemoglobin level before resuming dialysis and ABO blood type, while the risk of death was associated with comorbidities and residual eGFR.

## Introduction

Compared with other modalities of renal replacement therapies, kidney transplant is associated with improved quality of life, lower healthcare system costs, and longer life expectancy for patients living with advanced chronic kidney disease (CKD) [1–6]. In recent decades the outcomes in the first year after transplantation have substantially improved, graft loss (GL) due to acute rejection (AR) has become a low-frequency event, and the incidence of AR is lower than 20% [7, 8]. Despite this, long-term grafts and patient survival have not been followed in the same way, and GL due to chronic dysfunction and death with a functioning graft remain the main causes of long-term graft losses [9]. In emerging and developing countries, where transplantation has increased over the last few decades, death due to infections is the main cause of graft losses, while chronic dysfunction is the primary cause in countries with a high human development index [10–12].

Reinitiating dialysis after GL triples the risk of death, mostly in the first three months, which is considered a critical period [13], and the mortality rate is twice as high in patients starting dialysis for the first time in the natural evolution of CKD in naïve kidneys [2, 14]. Given the mortality risk, returning to dialysis can significantly affect the wait time for a new kidney transplant. In the US, for instance, GL is the third most prevalent cause of CKD among candidates on waiting lists [15, 16]. A previous transplantation is the strongest factor for anti-HLA antibody generation, with low odds of being matched with a compatible donor. Consequently, after GL, the likelihood of undergoing a new transplantation can be lower than that of death [17]. There are few studies exploring the long-term crude outcomes after GL, considering that most finished their observations when patients resumed dialysis [13]. Extending this follow-up period might demonstrate relevant data that can be embodied in the clinical approaches to this population.

Aside from a few reports, there are no studies evaluating long-term outcomes after GL including patients living in emerging and developing countries. This is relevant because the number of kidney transplantation in these nations, such as Brazil, has increased, and the epidemiological data from patients living in wealthy countries might not be extrapolated because they have different demographic characteristics than poorer countries. Thus, in this study, we evaluated the projected incidence of death and retransplantation after GL in a Brazilian single-center cohort and analyzed the variables related to each of these outcomes.

## Patients and methods

### Study design and population

This was a retrospective single-center cohort study conducted at the transplantation program at Hospital Israelita Albert Einstein in São Paulo, Brazil. São Paulo is the capital of São Paulo state and the most populous city and state in the country. Brazil has the largest public transplantation program in the world. Our hospital is a private hospital that provides philanthropic services through the Ministry of Health’s PROADI-SUS funding program. This study was approved by Research Ethics Committee, Hospital Israelita Albert Einstein, which can be contacted at cep@eisnstein.br. The approval number was CAAE 81185817.4.0000.0071 (included as a S1 and S2 Appendices). The need for consent was waived from the ethics committee.

**Fig 1:**
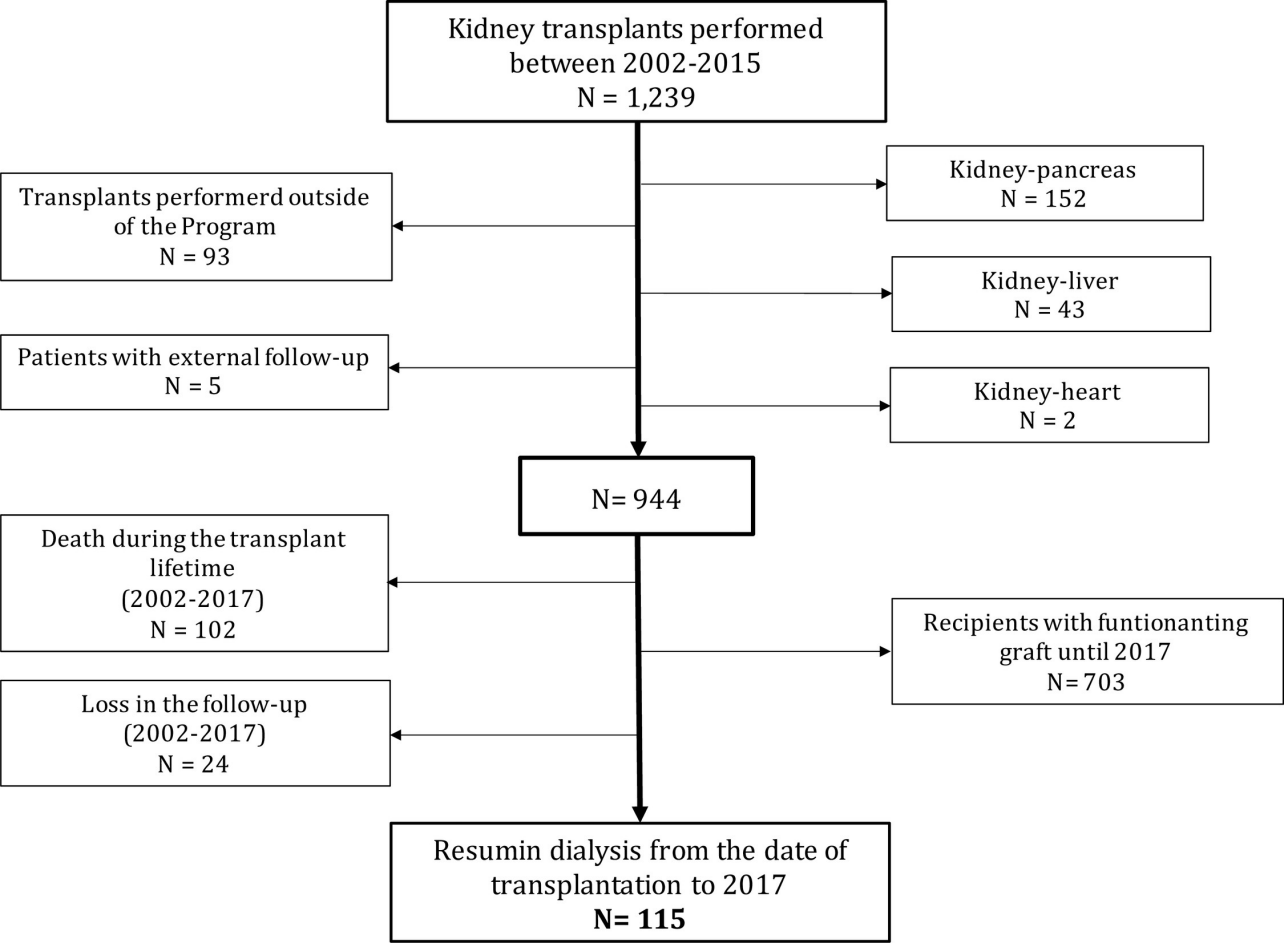
Flowchart of the population included in the cohort.

Patients who underwent kidney transplantation between 2002 and 2015 and resumed dialysis until 2017 were eligible for this cohort. Thus, all had the possibility of being followed for at least two consecutive years after transplantation. Other inclusion criteria and exclusion criteria were published previously [12], and they are summarized in the flow diagram depicted in Fig 1. Between 2002 and 2015, 1,239 transplantations were performed, and details concerning the patients who were excluded, as well as GL and death etiology were previously published too [12]. Additional details about the cohort composition are cited in S1 Appendix. Therefore, the current cohort (Fig 1) was composed of 115 recipients who survived transplantation and resumed dialysis due to graft failure (n = 92) or primary nonfunction (n = 23).

### Data source

The data on the time before GL were extracted from 2016 to 2017 from electronic medical records. Data after resuming dialysis were collected during 2019. The events that occurred at another healthcare service were monitored by a nurse responsible for information management (author: PRB). Through this monitoring, all of the patients or their relatives were contacted by telephone, and all of the data were verified and confirmed by the respective dialysis care centers, the office that coordinates the regional transplant waiting lists, or the National Transplantation System. In cases of death, a copy of the official death certificate issued by the Brazilian government was requested from the patient’s relatives for data confirmation.

### Outcomes, variables, definitions and follow-up after GL

The outcomes were death or retransplantation after GL. Graft loss was defined as resuming chronic dialysis or graft removal, whichever came first.

The variables were classified as three groups: demographic baseline, variables related to the time while the graft was functioning (transplant lifetime), and those related to resuming dialysis. In the first group, the recipient and donor characteristics were considered at the engraftment time. The following recipient characteristics were: age, gender, ethnicity, ABO blood type, CKD etiology, time in dialysis before transplant, and reactivity against a panel of lymphocytes (PRA). The following donor characteristics were considered: donor type (living or deceased), cause of brain death and if the expanded criteria were present in deceased donors, age, creatinine before donation, hypertension, and HLA matches in the ABDR HLA loci. To comparison, the era of transplantation was dichotomized in two periods: before 2009 and 2009 or later.

In the second group, the clinical characteristics were collected over the time while the recipients had functioning grafts, such as: type of immunosuppression; delayed graft function (DGF) defined as dialysis requirement in the first week after transplantation; acute rejection (cellular or antibody-mediated) proved by biopsy or clinically defined; CMV infection and post-transplant diabetes; time for GL and its etiology categorized by chronic rejection, acute rejection, thrombosis, recurrence of underlying renal disease, and others. The type of GL was categorized as a failure (when the recipients had a functioning graft loss) or primary absence of graft function (in cases of thrombosis and primary nonfunction) according to previously published data [12]. Details about immunosuppression approach and prophylaxis are presented in S2 Appendix.

In the third group, the variables related to resuming dialysis were: Charlson comorbidity index [18]; dialysis modality and access, such as arterio-venous (AV) fistula as access for hemodialysis, venous catheters, or peritoneal catheters for peritoneal dialysis; and biochemical parameters such as creatinine, hemoglobin, urea, potassium, ionic calcium, phosphorus, parathormone (PTH), serum albumin and C-reactive protein. The residual graft function was estimated by the CKD-epi formula, based on the last creatinine level available at the time when the patient had resumed to dialysis. Considering that we have a very mixed population in our country, we did not adjusted the CKD-epi for African ethnicity [19]. While the patients were undergoing dialysis (in a pre-transplant evaluation or on the waiting list), four additional variables were available: prednisone maintenance, graft nephrectomy, number of blood transfusions, and PRA.

Levels of hemoglobin, urea and potassium were collected on the same day when the patient had resumed to dialysis, until 7 days before and 14 or more days before in 69%, 16.9% and 14.1% of patients, respectively. Levels of calcium and phosphorous were collected on the same day when the patient had resumed to dialysis, until 7 days before and 14 or more days before in 54.6%, 20.2% and 25.2% patients, respectively. The median time when the PTH level was obtained was 30 (0; 158) days before the day when the patients had resumed to dialysis; 35% of the levels were obtained on the same day when the patients had resumed to dialysis. A patient was defined as with a AV fistula at the time when the graft loss. After GL no one patient changed the dialysis modality over the follow up.

Before 2006, PRA was measured by CDC. However, in this cohort only 3 patients had their PRA measured in that age. The others 83, it was calculated (cPRA) based on the Single Antigen issue using the frequency of HLA observed in the pool of donors in the city of São Paulo. The cut off for anti HLA was 2,000 mfi. In 19 patients, the PRA was collected on the same day or before patients had their graft lost: 5.2 (0.0; 0.25) months. In 67, it was collected 12.9 (4.7; 29.6) months after to resuming dialysis.

Patients who had GL started in-hospital dialysis and a few days later were transferred to a specialized service near their homes to follow their outpatient treatments. The clinical approaches for dialysis care, such as dose adjustment, control of CKD consequences, and treatment of the comorbidities, were managed by the dialysis team. In terms of immunosuppression, only low-dose prednisone (5 mg/day) was sustained for at least 6 months unless the patient underwent graft nephrectomy when immunosuppression was withdrawn. At the time when patients had resumed dialysis other immunosuppressive drugs different from prednisone were withdrawn.

Each patient was considered for retransplantation if a living donor was available; if not, they were included on the waiting list. Since 2002, kidney allocation has been based on ABO identity and the best matches in the ABDR HLA loci. Of note, candidates on the waiting list who have failed all possibilities for vascular access for hemodialysis and/or peritoneal failure are placed at the top of the list. Since January 2010, in the state of São Paulo (where the study was conducted), recipients of a previous non-kidney solid organ transplant or living kidney donors with stage 5 CKD are also placed at the top of the list.

### Statistical analysis

Continuous variables were summarized as the mean and standard deviation if they had a normal distribution; if not, they were presented as the median and interquartile range (first and third IQR). The normality was calculated by the Shapiro-Wilk test. Categorical variables are presented as frequencies and percentiles. All of the variables were compared one by one in two-tailed hypothesis tests, considering patients who underwent retransplantation versus those who did not and among patients who died versus those who did not after GL. Continuous variables were compared by student’s t-test or the Mann-Whitney U test in accordance with the normality distribution, while the categorical variables were compared using X^2^ or Fisher’s exact test depending on the estimated frequency in a two-way table.

To build the multivariable models, we selected variables that had better performance in the two-tailed comparison, arbitrarily defined as a P value equal to or lower than 0.10. We excluded from the model variables that had missing values and others that were collinear, independent of the P value. The models were run by Cox proportional hazard regression and the variables were filtered by backward steps. After the initial analysis, one variable with seven missing values, the serum phosphorus level when resuming dialysis, was included in a new model because it demonstrated relevant performance in the two-tailed comparison and we thought that it had a strong clinical relevance. In a sensitivity analysis, we imputed the median of the phosphorous missing values. Cumulative incidences of retransplantation and death were calculated by Kaplan-Meier tests and compared using the log-rank test. Statistical analyses were conducted using SPSS 26 (IBM, Armonk, NY, USA), and statistical significance was defined as P<0.05, with a 95% confidence interval (95% CI).

## Results

### The probability of retransplantation and death

**Fig 2:**
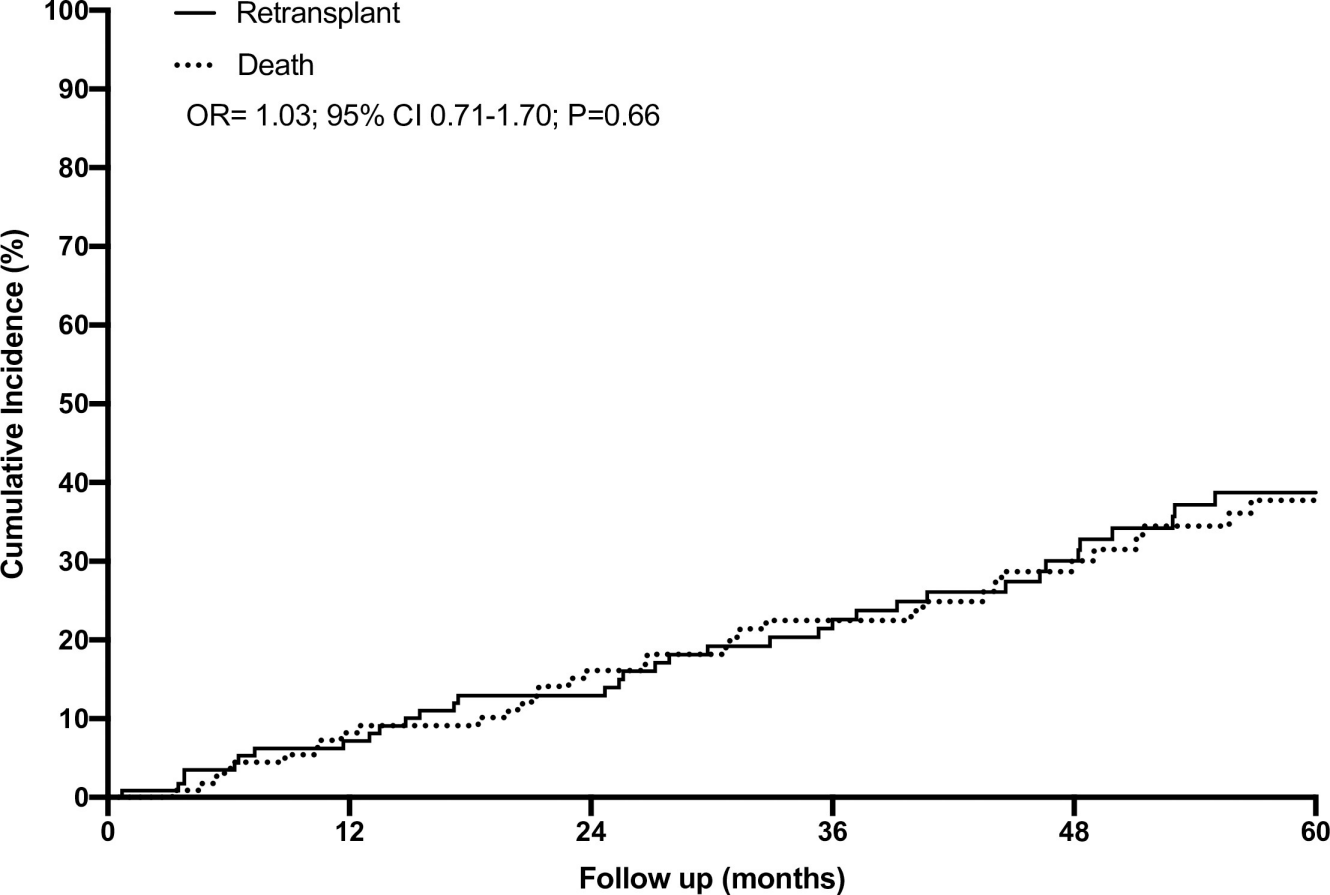
Cumulative incidence of retransplantation and death after graft loss.

The demographic baseline characteristics and clinical variables related to transplant lifetime and resuming dialysis are summarized in Table 1. The follow-up time after GL was 44.1 (21.4; 72.6) months. Over time, the incidence of retransplantation was 32.7 per 100 patient-years, corresponding a crude ratio of 37.4% in a median time of 35.5 months (14.8; 54.9). However, the incidence of death was 30.8 per 100 patient-years, corresponding to crude ratio of 33.4% in a median time of 30.7 months (12.4; 49.0). There was no difference in the projected 5-year probability of retransplantation or death (Fig 2): 38.7% for retransplantation (95% CI: 26.1%-51.2%) and 37.7% for death (95% CI: 24.9%-50.5%); odds ratio = 1.03 (95% CI: 0.71–1.70), P = 0.66.

**Table 1:** Demographic baseline characteristics and variables related to transplant lifetime and resuming dialysis in the total population and according to retransplantation status.

Variables	Total (N = 115)	RETRANSPLANTATION		P	Missing
		Yes (43)	No (72)		
**Demographic baseline characteristics**					
Age—years	40.6±14.8	37.2±12.2	42.4±16.0	0.10	0
Sex (male)–% (n)	59.1 (68)	65.1 (28)	55.6 (40)	0.31	0
Ethnicity (African-American)–% (n)	7.0 (8)	4.7 (2)	8.3 (6)	0.37	0
ABO blood type–% (n)				0.13	0
Type A ^(1)^	47.8 (55)	58.2 (25)	41.7 (30)		
Type O ^(2)^	41.7 (48)	30.2 (13)	48.6 (35)		
Type B	6.2 (7)	9.3 (4)	4.2 (3)		
Type AB	4.3 (5)	2.3 (1)	5.5 (4)		
CKD etiology–% (n)				0.09	0
Glomerulonephritis ^(3)^	27.8 (32)	39.5 (17)	20.8 (15)		
Unknown	20.9 (24)	23.2 (10)	19.4 (14)		
Hypertension	15.7 (18)	9.3 (4)	19.4 (14)		
Diabetes ^(4)^	13.0 (15)	4.7 (2)	18.1 (13)		
Urological	7.8 (9)	7.0 (3)	8.3 (6)		
Polycystic disease	7.0 (8)	4.7 (2)	8.3 (6)		
Others	7.8 (9)	11.6 (5)	5.7 (4)		
Time in dialysis–months	31.2 (17.0; 56.1)	28.3 (14.6; 51.5)	35.1 (17.9; 58.9)	0.56	0
PRA (before the transplant)–medians of %	0.0 (0.0; 4.0)	0.0 (0.0; 7.0)	0.0 (0.0; 5.2)	0.82	26
Previous engraftment–% (n)				0,98	0
0	92.2 (106)	95.4 (41)	90.3 (65)		
1	4.3 (5)	2.3 (1)	5.5 (4)		
2	3.5 (4)	2.3 (1)	4.2 (3)		
Transplantation period				0.99	0
Before 2009	61.7 (71)	38.0 (27)	62.0 (44)		
2009 or later	38.3 (44)	36.4 (16)	63.6 (28)		
Deceased donor–% (n)	56.5 (65)	44.2 (19)	63.9 (46)	0.04	0
Death due to cerebrovascular accident ^(5)^	62.1 (41)	60.0 (12)	63.0 (29)	0.81	0
Expanded criteria ^(5)^	16.9 (11)	36.1 (6)	10.9 (5)	0.07	0
Donor age—years	43.1±12.3	44.7±11.1	42.1±13.0	0.27	0
Donor creatinine—mg/dL	0.9 (0.7; 1.5)	0.90 (0.70; 1.50)	0.94 (0.73; 1.47)	0.56	0
Hypertension donor–% (n)	21.7 (25)	20.9 (9)	22.2 (16)	0.87	0
Mismatches (n)					0
MM A	1.15±0.64	1.16±0.67	1.14±0.61	0.85	
MM B	1.15±0.64	1.12±0.66	1.17±0.63	0.68	
MM DR	1.0 (0.0; 1.0)	1.0 (0.0; 1.0)	1.0 (0.0; 1.0)	0.37	
Sum of MM	3.12±1.56	3.19±1.64	3.08±1.52	0.73	
**Clinical variables related to transplant lifetime**					
Thymoglobulin induction–% (n)	60.9 (70)	53.5 (23)	65.3 (47)	0.21	0
Tacrolimus–% (n)	73.9 (85)	74.4 (32)	73.6 (53)	0.92	0
Mycophenolate–% (n)	78.3 (90)	79.1 (34)	77.8 (56)	0.87	0
DGF–% (n) ^(6)^	64.6 (42.6)	35.9 (17)	44.4 (32)	0.70	0
AR–% (n)					0
Cellular	55.7 (64)	58.1 (25)	54.2 (39)	0.68	
Antibody-mediated	24.3 (28)	16.3 (7)	29.1 (21)	0.12	
CMV–% (n)	50.4 (58)	44.2 (19)	54.2 (39)	0.30	0
Posttransplant diabetes–% (n)	12.7 (14)	9.8 (4)	14.5 (10)	0.47	5
Type of graft loss–% (n)				0.25	0
Failure	80.0 (92)	74.4 (32)	83.3 (60)		
Primary loss ^(7)^	20.0 (23)	25.6 (11)	16.7 (12)		
Etiology of graft loss–% (n)				0.43	0
Chronic rejection	40.0 (46)	34.9 (15)	43.0 (31)		
Acute rejection	18.3 (21)	11.6 (5)	22.2 (16)		
Thrombosis	17,4 (20)	20,9 (9)	15,3 (11)		
Recurrence	16.5 (19)	20.9 (9)	13.9 (10)		
Primary nonfunction	2.6 (3)	4.7 (2)	1.4 (1)		
Others	5.2 (6)	7.0 (3)	4.2 (3)		
Time to graft loss—months	38.0 (2.0; 85.0)	20.0 (2.0; 79.0)	41.5 (2.0; 86.7)	0.26	0
**Variables related to resuming dialysis**					
Charlson comorbidity index—points	3.0 (2.0; 5.0)	2.0 (2.0; 3.0)	3.5 (2.0; 6.0)	<0.001	
Historic of acute myocardial infarction—% (n)	10.4 (12)	4.7 (2)	13.9 (10)	0.12	
Heart failure—% (n)	15.6 (18)	16.3 (7)	15.3 (11)	0.89	
eGFR–mL/min/1.73 m^2^	7,2 (5,5; 9,2)	6.8 (5.2; 7.6)	8.3 (5.8; 10.3)	0.01	
Hemodialysis–% (n)	96.5 (111)	100 (43)	94.4 (68)	0.12	0
AV fistula access–% (n)	65.2 (75)	69.8 (30)	62.5 (45)	0.43	0
Hemoglobin—g/dL	9.20±1.80	9.56±2.15	8.98±1.53	0.09	0
Urea—mg/dL	128.8±56.1	130.8±55.8	127.7±56.6	0.78	2
Potassium—mEq/L	4.51±0.70	4.60±0.75	4.45±0.66	0.29	0
Ionic calcium—mmol/L	1.18±0.13	1.18±0.08	1.18±0.15	0.91	5
Phosphorus—mg/dL	5.18±1.45	5.31±1.49	5.09±1.43	0.44	7
PTH intact—pg/dL	179.4 (84.7; 310.6)	179.4 (62.9; 319.0)	180.1 (93.6; 300.9)	0.80	38
Serum albumin–g/dL	3.20±0.73	3.11±0.83	3.25±0.67	0.52	61
C-reactive protein–mg/dL	30.3 (10.0; 86.1)	30.3 (8.7; 140.3)	29.6 (10.0; 75.1)	0.68	25
Graft nephrectomy–% (n)	53.9 (62)	65.1 (28)	47.2 (34)	0.06	0
After late loss—% (n)	37.4 (43)	46.5 (20)	31.9 (23)	0.12	
Prednisone–% (n)	21.7 (46)	30.2 (13)	45.8 (33)	0.10	0
Blood transfusion after graft loss–% (n)	35.0 (34)	40.0 (16)	31.6 (18)	0.39	18
PRA after graft loss—medians of % ^(8)^	47.0 (0.0; 83.0)	49.0 (0.0; 75.0)	38.5 (0.0; 88.0)	0.94	26
PRA ≥ 50%—% (n)	47.2 (42)	48.7 (19)	46.0 (23)	0.80	

Blood type A (1): P = 0.09

Blood type O (2): P = 0.05

CKD etiology: (3) P = 0.03 for glomerulonephritis and (4) P = 0.04 for diabetes

Death due to cerebrovascular and expanded criteria donor (5): only for deceased donors (n = 65)

DGF (6): considered for all recipients, as those from deceased donors, as living donors

Primary losses (7): accounting for loss due to thrombosis and primary nonfunction. Categorizing the primary losses in thrombosis and primary nonfunction (PNF), there were no differences between the frequency of events according to retransplantation status. Nineteen patients lost their grafts due to thrombosis (8 who were retransplanted and 11 who were not), and 3 lost their grafts due to PNF (2 who were retransplanted and 1 who was not); P = 0.57

PRA (8): in 3 patients PRA was measured by CDC (before 2007); in 89 it was calculated (cPRA) based on the Single Antigen and in the frequency of HLA observed in the pool of donors in the city of São Paulo.

CKD: chronic kidney disease; PRA: panel reactivity against lymphocyte antigen; MM: mismatch; DGF: delayed graft function; AR: acute rejection; CMV: cytomegalovirus infection; eGFR: estimated glomerular filtration rate; AV: arteriovenous; PTH: parathormone.

### Characteristics associated with retransplantation

The variables were compared between the patients who underwent retransplantation (n = 43) and those who did not (n = 72); this comparison is detailed in Table 1. The incidence of CKD due to glomerular diseases was higher (39.5 vs 20.8%, P = 0.03) in the retransplanted patients, while the incidences of CKD due to diabetes (4.7 vs 18.1%, P = 0.04) and deceased donors (44.2 vs 63.9%, P = 0.04) were lower. There were no differences in the variables related to transplant lifetime, including the time to GL. The frequency of retransplant status according to transplant era, dichotomized in before 2009 or later, was not different in both eras: 38% < 2009 vs. 36.4% ≥ 2009, P = 0.99. At the time of resuming dialysis, patients who underwent retransplantation presented a lower Charlson comorbidity index (2.0 vs. 3.5 score, P<0.001), as well as a lower eGFR (6.8 vs. 8.3 ml/min/1.73 m^2^, P = 0.01). The trend toward a higher frequency of graft nephrectomy was observed in patients who underwent retransplantation: 65.2 vs 47.2%, P = 0.06. However, there was no difference between the groups when we considered only nephrectomies carried out after late graft loss (P = 0.12, excluding nephrectomy due to thrombosis).

Table 2 shows the results of the sixth run of the Cox regression for the likelihood of retransplantation, which was higher when patients resumed dialysis with higher hemoglobin levels (HR = 1.22; 95% CI = 1.04–1.43; P = 0.01), while it was lower in those with type O blood (HR = 0.48; 95% CI = 0.25–0.93; P = 0.03).

**Table 2:** Cox regression for the probability of retransplantation.

Variables	B	HR	95% CI		P
			Lower	Upper	
Blood type O ^(1)^	-0.735	0.48	0.25	0.93	0.03
Hemoglobin level ^(2)^	0.200	1.22	1.04	1.43	0.01

(1) Blood type O vs. other blood types

(2) Hemoglobin level at the return to dialysis

Variables included in the modeling: age, ABO blood type, the etiology of chronic kidney disease, the type of donor (living vs. deceased), Charlson comorbidity index, eGFR and hemoglobin level at the time of resuming dialysis, prednisone sustained after graft loss and graft nephrectomy

Among the variables that reached a P-value ≤ 0.10 in the one-by-one analysis, expanded criteria donor was not included in this modeling because this variable is observed only in deceased donors.

### Influence of ABO blood type and HLA matches

Among the patients who underwent retransplantation (n = 43), the first transplant was from a living donor in 55.8% (n = 24), while the subsequent transplant was from a deceased donor in two-thirds (n = 16) and from a living donor in the others (n = 8). However, among those in whom the first transplant was from a deceased donor (n = 19; 44.2%), the second was from a deceased donor in all (Table 3A). Time to retransplantation tended to be shorter in those who received the subsequent kidney from a living donor: 21.0 (8.1; 34.3) vs. 39.2 (15.5; 55.0) months, P = 0.07. Table 3B details the transplantation and retransplantation frequency according to the ABO blood type. Of note, the retransplant of type O recipients were mostly from deceased donors, while the retransplantation frequency of type A and B living donors was 3 times higher. Although the HLA-matched compatibility in the previous transplant did not influence the odds of retransplantation, the ABDR mismatch frequency according to the type of donor, as in the previous and the subsequent transplant, are detailed in S1 and S2 Tables.

**Table 3:** Frequency of transplanted and retransplanted recipients according to donor type and ABO blood type.

3A. Frequency of transplantations according to donor type.								
**1**^**st**^ **donor**	**N**	**%**	**2**^**nd**^ **donor**	**N**	**%**			
Living	24	55.80%	Living	8	33.30%			
			Deceased	16	66.70%			
Deceased	19	44.20%	Living	0	0%			
			Deceased	19	100%			
3B. Frequency of transplantations according to ABO blood type.								
Blood type	**O**		**A**		**B**		**AB**	
Total, n = 115 (%)	48 (41.7%)		55 (47.8%)		7 (6.1%)		5 (4.3%)	
Retransplantation, n = 43 (%)	13 (27.1%)		25 (45.4%)		4 (57.1%)		1 (20.0%)	
Donor type	Living	Deceased	Living	Deceased	Living	Deceases	Living	Deceased
	1 (7.7%)	12 (92.3%)	6 (24.0%)	19 (76.0%)	1 (25.0%)	3 (75.0%)	0 (0%)	1 (100%)
								

Time to retransplantation: 21.0 (8.1; 34.3) months in those who received the subsequent kidney from a living donor and 39.2 (15.5; 55.0) months in those who received the subsequent kidney from a deceased donor; P = 0.07.

### Characteristics associated with the risk of death

All of the variables were compared one by one (Table 4) among the patients who died after GL (n = 39) versus those who survived (n = 76). The patients who died were older at the time of transplantation (48.0 vs 36.9 years, P<0.001) and more frequently had CKD due to diabetes (30.7 vs 3.9%, P<0.001). Moreover, they had a higher frequency of grafts from deceased donors (69.2 vs 50.0%, P = 0.05) and therefore a higher level of serum creatinine before donation (P = 0.03). Considering the transplant lifetime variables, the incidence of DGF was higher among the patients who died (59.0 vs 34.2%, P = 0.02) and a trend of a lower incidence of cellular AR (43.5 vs 61.8%, P = 0.06), in addition to earlier episodes (0.50 vs 8.20 months, P = 0.03), was noted among the patients who died. Here, we analyzed the death rate according to transplant era (before 2009 or later) and it was the same in both eras (35.2 vs. 31.8%, P = 0.84). When resuming dialysis, patients who died presented a significantly higher Charlson comorbidity index (5.0 vs. 2.0 score, P<0.001) and a higher eGFR (8.8 vs. 6.8 ml/min/1.73 m^2^, P = 0.001). There were no significant differences in the modality of renal replacement therapy; however, the frequency of patients who started hemodialysis after graft loss by AV fistula access compared to hemodialysis using venous catheters or peritoneal dialysis was lower in the patients who died (51.3 vs 72.4%, P = 0.02). They also had lower phosphatemia (4.49 vs 5.50 mg/dL, P = 0.001).

**Table 4:** Demographic baseline characteristics and variables related to transplant lifetime and resuming dialysis in patients who died versus those who survived after graft loss.

Variables	Death		P
	Yes (39)	No (76)	
**Demographic baseline characteristics**			
Age—years	48.0±15.1	36.9±13.3	<0.001
Sex (male)–% (n)	59.0 (23)	59.2 (45)	0.98
Ethnicity (African-American)–% (n)	7.7 (3)	6.6 (5)	0.55
ABO blood type–% (n)			0,98
Type A	48.8 (19)	47.4 (36)	
Type O	41.0 (16)	42.1 (32)	
Type B	5.1 (2)	6.6 (5)	
Type AB	5.1 (2)	3.9 (3)	
CKD etiology–% (n)			<0.001
Glomerulonephritis	20.5 (8)	31.6 (24)	
Unknown	7.7 (3)	27.6 (21)	
Hypertension	10.3 (4)	18.4 (14)	
Diabetes ^(1)^	30.7 (12)	3.9 (3)	
Urological	12.8 (5)	5.3 (4)	
Polycystic disease	10.3 (4)	5.3 (4)	
Others	7.7 (3)	7.9 (6)	
Time in dialysis–months	39.9 (17.9; 59.9)	28.3 (15.6; 55.3)	0.44
PRA (before the transplant)–medians of %	0.0 (0.0; 0.0)	0.0 (0.0; 10.0)	0.18
Previous engraftment–% (n)			0,09
0	84.6 (33)	96.0 (73)	
1	7.7 (3)	2.6 (2)	
2	7.7 (3)	1.4 (1)	
Transplantation period			0.84
Before 2009	35.2 (25)	64.8 (46)	
2009 or later	31.8 (14)	68.2 (30)	
Deceased donor–% (n)	69.2 (27)	50.0 (38)	0.05
Death due to cerebrovascular accident ^(2)^	63.0 (17)	61.5 (24)	0.91
Expanded criteria ^(2)^	7.4 (2)	23.7 (9)	0.08
Donor age—years	42.2±11.8	43.5±12.6	0.58
Donor creatinine—mg/dL	1.00 (0.80; 1.70)	0.9 (0.70; 1.35)	0.03
Hypertension donor–% (n)	25.6 (10)	19.7 (15)	0.47
Mismatches (n)			
MM A	1.15-±0.54	1.14±0.69	0.94
MM B	1.31±0.61	1.07±0.64	0.05
MM DR	1.00 (0.00; 2.00)	1.00 (0.00; 1.00)	0.71
Sum of MM	3.33±1.50	3.01±1.59	0.30
**Clinical variables related to transplant lifetime**			
Thymoglobulin induction–% (n)	69.2 (27)	56.6 (43)	0.19
Tacrolimus–% (n)	82.1 (32)	69.7 (53)	0.15
Mycophenolate–% (n)	76.9 (30)	78.9 (60)	0.80
DGF–% (n) ^(3)^	59.0 (23)	34.2 (26)	0.02
AR–% (n)			
Cellular	43.6 (17)	61.8 (47)	0.06
Antibody-mediated	30.8 (12)	21.1 (16)	0.25
Time to AR–months	0.5 (0.20; 6.55)	8.20 (0.50; 36.2)	0.03
CMV–% (n)	61.5 (24)	44.7 (34)	0.09
Posttransplant diabetes–% (n)	15.8 (6)	11.1 (8)	0.34
Type of graft loss–% (n)			0.17
Failure	87.2 (34)	76.3 (58)	
Primary loss ^(4)^	12.8 (5)	23.7 (18)	
Etiology of graft loss–% (n)			0.71
Chronic rejection	48.7 (19)	35.5 (27)	
Acute rejection	17.9 (7)	18.4 (14)	
Thrombosis	10.3 (4)	21.1 (16)	
Recurrence	15.4 (6)	17.1 (13)	
Primary nonfunction	2.6 (1)	2.6 (2)	
Others	5.1 (2)	5.3 (4)	
Time to graft loss—months	43.0 (3.0; 86.0)	32.0 (1.2; 82.7)	0.29
**Variables related to resuming dialysis**			
Charlson comorbidity index—points	5.0 (3.0; 6.0)	2.0 (2.0; 3.0)	<0.001
Acute myocardial infarction—% (n)	17.9 (7)	6.6 (5)	0.10
Heart failure—% (n)	17.9 (7)	14.5 (11)	0.63
eGFR–mL/min/1.73 m^2^	8.8 (6.5; 11.5)	6.8 (5.2; 8.3)	0.001
Hemodialysis–% (n)	92.3 (36)	98.7 (75)	0.11
AV fistula access–% (n)	51.3 (20)	72.4 (55)	0.02
Hemoglobin—g/dL	9.10±1.54	9.25±1.93	0.70
Urea—mg/dL	126.9±61.2	129.8±53.6	0.80
Potassium—mEq/L	4.50±0.72	4.51±0.67	0.89
Ionic calcium—mmol/L	1.20±0.09	1.17±0.14	0.30
Phosphorus—mg/dL	4.49±1.31	5.50±1.41	0.001
Serum albumin–g/dL	3.26±0.69	3.16±0.78	0.61
C-reactive protein–mg/dL	33.3 (10.7; 57.7)	29.2 (8.0; 135.0)	0.80
PTH intact—pg/dL	180.1(107.7; 287.7)	179.4 (62.9; 319.1)	0.26
Graft nephrectomy–% (n)	41.0 (16)	60.5 (46)	0.05
After late loss—% (n) ^(5)^	30.8 (12)	40.8 (31)	0.29
Prednisone–% (n)	51.3 (20)	34.2 (26)	0.08
Blood transfusion after graft loss–% (n)	23.1 (6)	39.4 (28)	0.13
PRA after graft loss—medians of % ^(6)^	7.50 (0.00; 76.0)	53.0 (0.00; 85.5)	0.14
PRA ≥ 50%—% (n)	30.0 (6)	52.2 (36)	0.08

CKD etiology: (1) P <0.001 for diabetes

Death due to cerebrovascular accident and expanded criteria donor (2): only for deceased donors (n = 65)

DGF (3): considered for all recipients, as those from deceased donors, as living donors

Primary losses (4): accounting loss due to thrombosis and primary nonfunction

Graft nephrectomy late loss (5): excluding graft nephrectomy due to early thrombosis

PRA (6): in 3 patients, PRA was measured by CDC (before 2007); in 89 it was calculated (cPRA) based on the Single Antigen issue using the frequency of HLA observed in the pool of donors in the city of São Paulo.

CKD: chronic kidney disease; PRA: panel reactivity against lymphocyte antigen; MM: mismatch; AR: acute rejection DGF: delayed graft function; CMV: cytomegalovirus infection; eGFR: estimated glomerular filtration rate; AV: arteriovenous; PTH: parathormone.

The main cause of death was infection, with 35.9% of deaths being due to sepsis with the primary site of infection being well defined and 15.4% due to multiple organ and system dysfunction (MODS) without a primary site of infection being well defined or documented. The following causes were cardiovascular events (15.4%) and cancer (10.2%). The deaths due to cancer were based on 4 events: melanoma, adenocarcinoma of the lung, carcinoma of bladder and sarcoma of unknown primary site. In 12.7% of deaths it was not possible to establish their causes, while cirrhosis, trauma, acute complications of diabetes and uremia occurred in one patient (2.6%) each one. This last was the cause of death in a highly sensitized patient (PRA 100%) with dialysis access failure.

In the Cox regression, the risk of death was significantly associated with two variables at the time to resuming dialysis (Table 5): CCI and eGFR. The risk of death was increased by 37% for each point on the CCI (HR = 1.37; 95% CI = 1.19–1.50; P<0.001), and by 14% for each mL/min/1.73 m^2^ increase in eGFR (HR = 1.14; 95% CI = 1.05–1.25; P = 0.002). The trend toward a lower risk of death in patients who had resumed to dialysis using AV fistula access was observed (HR = 0.50; 95% CI 0.25–1.02; P = 0.06), while higher risk seems to be associated with the number of previous engraftment (HR = 2.01; 95% CI 0.99–4.07; P = 0.05). These results were not affected when they were adjusted for age.

**Table 5:** Cox regression for risk of death.

Variables	B	HR	95% CI		P
			Lower	Upper	
AV fistula access ^(1)^	-0.686	0.50	0.25	1.02	0.06
Number of previous engraftment ^(2)^	0.698	2.01	0.99	4.07	0.05
Residual eGFR ^(3)^	0.135	1.14	1.05	1.25	0.002
Charlson comorbidities index ^(4)^	0.317	1.37	1.19	1.50	<0.001

(1) Resuming dialysis by vascular graft access vs. venous catheter and peritoneal catheter

(2) Included as an ordinary variable: no previous engraftment, 1 or 2 previous engraftments

(3) Each 1 mL/min/1.73 m2 of eGFR at the time to resuming dialysis, estimated by CKD-epi

(4) Each 1 point in the index at the time to resuming dialysis

Variables included in this modeling (modeling 1): number of previous kidney engraftments, type of donor (living vs. deceased), number of mismatches in locus B; delayed graft function; cellular AR; infection due to cytomegalovirus; AV fistula access; residual eGFR and Charlson comorbidities index at the time to resuming dialysis; graft nephrectomy due to chronic failure (excluding those due to primary loss).

Among variables that reached P-value ≤ 0.10 in the one-by-one analysis the following were not included: expanded criteria donor owing to this variable is observed only in deceased donors; levels of creatinine in donor, considering that this is a colinear variable with donor type; time to AR since it is not possible to impute values for recipients who did not have this event; and prednisone sustained after graft loss because all patients who had their graft removed after GL had the prednisone withdrew, thus this variable was collinear with graft nephrectomy. Furthermore, two other variables were not included in this modeling 1: age and etiology of chronic kidney disease (diabetes vs. others), considering the collinearity effect, since both were the most important variables that had defined the Charlson comorbidity index.

Backward stepwise removal of variables (modeling as described above): delayed graft function (step 2), donor type (step 3), number of mismatches in locus B (step 4), graft nephrectomy (step 5), infection by cytomegalovirus (step 6), and cellular AR (step 7). Moreover, the final results were not affected when model 1 was adjusted for age (model 2), and graft nephrectomy due to chronic failure was replaced by overall graft nephrectomy (model 3).

AR: acute rejection; AV: arteriovenous; eGFR: estimated glomerular filtration rate.

Because there was a strong difference in phosphatemia at the time before resuming dialysis among patients who died compared to those who survived, we ran an extra Cox regression model including this variable; however, the final result was exactly the same which was observed in the first modeling. At this time, phosphatemia was removed from the modeling in the third step (by backward modeling).

### Nephrectomy

The graft was removed from 62 patients (53.9%) and graft removal was more frequent among those who survived after GL: 60.5 vs 41.0%, P = 0.05 (Table 4). In 19 patients, graft nephrectomy was performed due to graft vascular thrombosis attributed to postoperative mechanical etiology, and only one was due to late thrombosis in a functioning graft 290 days after transplantation. In the others, transplant nephrectomy was performed in patients with graft failure an average of 30.5 months (0.17; 87.3) after transplantation and 120 days (0.0; 546.0) after GL. Having considered that are quite different with respect to the inflammatory-associated status between patients who underwent graft nephrectomy due to early thrombosis and those who underwent graft nephrectomy for other reasons, we analyzed the frequency of late nephrectomy among patients who died and those who did not. This frequency was lower among patients who died; however, this difference was not significant: 30.8% vs. 40.8%, P = 0.29.

## Discussion

Despite robust advances in short-term outcomes after kidney transplant, a consequent improvement in the long-term results has not been achieved [9]. Generally, the monitoring of the outcomes after kidney transplantation is stopped when the recipient returns to dialysis. Extending this follow-up can provide relevant information for clinical approaches in transplant patients, and the transition time between GL and resuming dialysis should be considered as a critical period after transplantation. The risk of death due to all causes in patients after kidney GL, for instance, is three times higher than that observed in patients with functioning grafts [20, 21], and returning to dialysis is related to a 47% higher risk of death due to cardiovascular disease and more than twice that due to infectious events [22]. Most epidemiological data regarding this critical period, as well as outcomes after GL, are from studies conducted in developed countries [13, 15, 23, 24]. However, the number of kidney transplants in emerging and developing countries has increased over the last few decades, and data from this population are scarce. Although this study was conducted at a single center, to the best of our knowledge it is the first to evaluate long-term outcomes after resuming dialysis in an emerging country.

Herein the probability of death after GL was not higher than that retransplantation. As previously discussed, returning to dialysis is a critical period in the lifetime of the transplant recipient. The mortality rate increases and some factors seem to be associated with the overall outcomes, such as age, underlying disease, cardiovascular condition, residual kidney function, and dialysis access type, among others [13, 20, 21, 25, 26]. Despite the small cohort, we found some variables related to mortality after GL.

The most important factor associated with the risk of death was the recipient’s burden of comorbidities at the time of resuming dialysis, assessed by the CCI. It is important to point out that the index was most impacted by the overall presence of CKD (mainly due to diabetes mellitus) and age. Diabetes mellitus has been widely recognized as one of the strongest factors associated with long-term mortality and it is equally associated with patients diagnosed with early stages of CKD and with those receiving renal replacement therapy [27, 28]. In an explorative analysis by the United States Renal Data System (USRDS) between 1995 and 2003 of stable patients on the waiting list for a kidney transplant, other stable kidney transplant recipients, and a third group of patients undergoing their second round of dialysis after GL, a higher mortality rate was associated with age and diabetes mellitus [24]. It is important to note that these patients can develop diabetes after transplant; however, diabetes as the etiology of CKD mostly impacts CCI, considering that in these patients we have to consider the target organ damage in diabetes, which is associated with extra points on the comorbidity index.

Another factor that could impact the risk of death is the number of previous kidney transplants, although less than 10% of the patients had previous transplantation. In the multivariable model, each prior engraftment tended to double the risk of mortality in the subsequent transplantation, which is a relevant point because the number of failed transplants has increased over the last few decades [15, 16, 29–31]. In contrast to this observation, in an interesting study based on the United Network for Organ Sharing (UNOS) database that included more than 17,000 retransplanted patients, survival was not different between a group of primary renal transplant patients and those who had undergone retransplantation [32].

The median of the residual function assessed by estimated GFR was 7.2 mL/min/1.73 m^2^, and it was slightly higher (but statistically significant) among patients who died after GL (8.8 vs. 6.8, P = 0.001). Each 1 mL/min/1.73 m^2^ in eGFR increased the risk of death by 14%. Although this result seems to be controversial, it has been observed in other cohorts. Earlier return to dialysis, defined as resuming dialysis when eGFR is higher than 10 mL/min/1.73 m^2^, seems to be associated with higher risk of mortality. Using a propensity score to evaluate the risk of death after transplant failure, Molnar et al demonstrated that the earlier return to dialysis was associated with diabetes mellitus (OR = 1.75) and peripheral vascular disease (OR = 3.55), two clinical conditions traditionally related with higher mortality [33]. Having examined more than 4,700 patients from the United States Renal Data System (USRD) whose transplants failed, Gill et al observed that each 1 mL/min/1.73 m^2^ higher eGFR at dialysis reinitiation increased the risk of death by 4% [25]. In the same way, in another large American cohort, Brar et al showed that eGFR < 10 mL/min/1.73 m^2^ at time of resuming dialysis reduced the risk of death by 17%. According to them, higher GFR alone would not be a good predictor of outcomes in patients who had resumed to dialysis, however it could be a marker of patients who are more unwell and they would have to be transitioned more quickly, mainly due to the presence of congestive heart failure, ischemic heart disease and cerebrovascular disease [34].

Age, diabetes, and previous transplantation are nonmodifiable, but it is possible that other factors could be better controlled, reaching a potential reduction in mortality rates after resuming dialysis. It is possible that controlling comorbidities related to CKD in transplant patients, such as blood pressure, anemia, secondary hyperparathyroidism, dialysis modality, and others, could be related to better outcomes, although there is no robust evidence based on controlled clinical trials demonstrating these benefits. For instance, it is unclear whether the dialysis modality can impact long-term outcomes in patients with GL. Several studies with different follow-up periods found no differences in mortality between hemodialysis and peritoneal dialysis [31, 35, 36]. However, in our cohort, resuming dialysis using AV fistula access, in contrast to vascular or peritoneal catheters, reduced the risk of death by 50%, although this result was not statistically significant in the multivariable modeling (P = 0.06). It is important to emphasize that only 3.5% of patients resume peritoneal dialysis, so it is likely that this result highlights the difference between the type of vascular access. Furthermore, other authors suggested that timely vascular graft construction may be associated with lower mortality [24]. Of note, many transplant patients maintain their vascular access function during the lifetime of the transplant. However, starting dialysis with adequate access might reflect the quality of clinical management based on CKD management targets [24, 25, 34]. Evidence has demonstrated that CKD management of transplant patients seems to be worse than that in nontransplanted patients [26, 37–41]. For example, in advanced CKD, hypertension and anemia were less likely to be treated in transplant patients [37], and when the clinical practice targets were measured, they were less likely to be met in patients with failed transplants [31]. The same scenario was found when transplanted patients were compared to patients already on dialysis: transplant patients had worse blood pressure control, higher serum phosphorus, lower bicarbonate, and lower hemoglobin [38].

Interestingly, phosphatemia was higher in patients who survived after resuming dialysis. Of note, in both, the averages were in the range recommended by best practices; however, among patients who did not die, the level was slightly high. This result should be carefully considered, primarily because we had a small number of at-risk patients, and these values each corresponded to the last available measurement time before patients had resumed dialysis. However, this exploratory result could lead to a very interesting hypothesis: if this biomarker was a clinical target, would levels closer to the upper limit be best practice? Considering that phosphatemia has a U- or J-shaped curve for mortality in patients with CKD [40–44], could a slightly higher phosphorus level (even on the target) be a marker of better nutrition? Actually, it seems that low and high serum phosphorus levels may be associated with mortality after kidney failure [45]. Hyperphosphatemia in transplant patients with advanced CKD occurs in approximately 20%, which is similar to patients nontransplanted who are managed in the pre-dialysis period [37], although the frequency of patients receiving phosphate chelation treatment is three times higher [37, 40]. To explore better this interaction, we used a sensitivity analysis imputing the median for missing values, however, we did not find an independent and significant association between phosphorus and mortality.

In the present study, AR episodes occurred earlier in the recipients who died: 0.5 vs 8.2 months. It is known that late events are primarily associated with low immunosuppression exposure due to clinical decisions or lower patient adherence [45, 46]. It is important to note that a low dose of immunosuppression (prednisone) was sustained after GL, on the other hand, when patients had their graft removed, any immunosuppression was withdrawn. The role of graft nephrectomy is an important point to be explored in studies designed to evaluate outcomes after GL. Early graft nephrectomy is indicated in cases of primary nonfunction due to thrombosis, which is associated with abrupt withdrawal of immunosuppression. Nevertheless, when GL is due to chronic dysfunction, immunosuppression is routinely maintained for months. Late graft nephrectomy after GL frequently occurs in patients with uncontrolled pain, hematuria, or fever or in cases of repeated urinary infections [47]. It is generally thought that, even in asymptomatic patients, graft maintenance may be associated with worse dialysis outcomes. Ayus et al. evaluated more than 10,000 kidney transplant patients who resumed dialysis and had a graft nephrectomy frequency of 31.5%, demonstrating that nephrectomy was associated with a 32% reduction in the risk of dying of any cause [48]. It is generally believed that early graft nephrectomy after GL can reduce the impact of inflammatory status in these patients and improve responsivity to anemia treatment and albumin levels, all markers of mortality in dialysis patients [49, 50]. In our cohort, all of late nephrectomy were carried out due to pain, hematuria or, less commonly due to fever. We have never indicated the nephrectomy preemptively.

Graft nephrectomy has not been associated with the probability of retransplantation. Instead, current evidence has demonstrated that graft nephrectomy increases the risk of sensitization, impacting the likelihood of retransplantation and even causing unfavorable outcomes [47, 51–53]. In a meta-analysis of 20 trials, graft maintenance reduced the risk of GL in a subsequent transplantation for 3 years (52% reduction) and 5 years (35% reduction) after the new transplant. In addition to the increase in the risk of sensitization and AR, graft removal increased the 5-year mortality risk after a subsequent transplantation by 82% [51].

Moreover, some studies found an association between graft nephrectomy and anti-HLA antibody development [53, 54]. It was previously considered that failed grafts had filter functions, absorbing circulating anti-HLA, although most recent evidence using more sensitive methodologies to assess anti-HLA has refuted this hypothesis [21]. It is possible that immunosuppression withdrawal after graft nephrectomy is the main cause of anti-HLA production [54, 55]. In our cohort, we failed to demonstrate an association between graft nephrectomy and retransplantation odds, likely due to the small number of patients, although nephrectomy was more frequent in the group of retransplantation patients (65.1 vs 47.2%). However, two other variables were significantly associated with the likelihood of subsequent retransplantation: hemoglobin level and ABO blood type.

The hemoglobin level had a positive association with the probability of retransplantation, which might be associated with better clinical management of these patients. Some anemic patients are more susceptible to blood transfusion and HLA sensitization. We measured the number of blood transfusions and PRA after resuming dialysis. Unfortunately, there were many missing values in these two variables (18 and 26, respectively), making further analyses impossible. The association with blood type O and low probability of retransplantation was clearer in the context of our transplantation system. Although one criterion for kidney allocation from deceased donors is blood type, there are few centers that perform HLA and ABO-incompatible transplants in Brazil. Of note, compared with other ABO blood types, type O decreased the likelihood of retransplantation by 52% (P = 0.03). The vast majority (92.3%) of retransplants were from deceased donors, considering that only one ABO-compatible living donor was available for the previous transplantation. Overall, 25% of type A and B patients received their retransplant from a living donor because they were able to receive a graft from a type A/B or O donor.

There are several limitations to this study. First, it was a historic cohort conducted at a single center. Consequently, the number of patients was low, and the results could not be extrapolated to other groups of patients, even under similar conditions, such as transplantation programs in emerging countries. Moreover, because this was a retrospective study, some variables had many missing values, mainly during the critical patient lifetime period, which is the time to resuming dialysis. Finally, the patients who resumed dialysis were treated by other teams, different from the transplant center, so there were variations in the clinical approaches to each patient, which makes it impossible to analyze the central factors associated with the risk of death and the probability of retransplantation. Notwithstanding, to date, this is the first study exploring outcomes after graft failure in an emerging country, and even with these limitations, some results can be useful for future clinical decisions and forthcoming prospective studies.

In conclusion, in this cohort, the probability of death was similar to that retransplantation 5 years after kidney GL. To the best of our knowledge, this is the first study evaluating long-term crude outcomes in patients who resumed dialysis after GL in an emerging country. There were significant variables associated with the probability of retransplantation, such as hemoglobin level when dialysis was resumed and ABO blood type, while the variables related to the risk of mortality included the recipient’s burden of comorbidities, residual eGFR, and possibly dialysis access type.

## Supporting information

S1 Appendix Inclusion and exclusion criteria and details about the cohort. (DOCX)

S2 Appendix Variables of interest, immunosuppression and prophylaxis approaches. (DOCX)

S1 Table Median of mismatches in ABDR *loci* in the first and in the second transplant. (DOCX)

S2 Table Median of mismatches in ABDR *loci* in the first and in the second transplant according to donor type (living vs. deceased). (DOCX)

## Abbreviations

CKD: Chronic kidney disease
GL: Graft loss
HLAs: Human leucocyte antigens
AR: Acute rejection
IQR: Interquartile range
CI: Confidence interval
OR: Odds ratio
CMV: Cytomegalovirus
DGF: Delayed graft function
MM: Mismatch
PRA: Panel reactivity against leucocyte antigen
AV: Arteriovenous
PTH: Parathormone
HR: Hazard ratio
