# Supplementary material for: Long-term outcomes after kidney transplant failure and variables related to risk of death and probability of retransplant: Results from a single-center cohort study in Brazil
Source: PLoS One. 2021 Jan 20;16(1):e0245628. doi: 10.1371/journal.pone.0245628 (PMC7816974; doi:10.1371/journal.pone.0245628)
Supplement: S1 Appendix — (DOCX) [file pone.0245628.s001.docx]

**APPENDIX 1: Inclusion and exclusion criteria and details about the cohort.**

Patients who underwent a kidney transplant between 2002 and 2015 and resumed dialysis until 2017 were eligible for this cohort. Thus, all had the possibility of being followed for at least two consecutive years after transplantation. In addition to the transplant year, other inclusion criteria were any age at the transplant time and grafts from any kind of donor. The exclusion criteria were as follows: kidney transplant recipients combined with another solid organ (simultaneous with the pancreas, liver, or heart) and transplants performed outside the PROADI-SUS program; that is, those with supplementary or private insurance as a source of funding, since their follow-up after transplantation was conducted independent of the aforementioned program. After returning to dialysis, the historic cohort was followed until 2019; thus, all of the patients had the possibility of being exposed to the risk for at least two consecutive years after GL.

Between 2002 and 2015, 1,239 transplants were performed; 197 were excluded because the recipients received a kidney combined with another solid organ (2 heart-kidney, 43 liver-kidney, and 152 pancreas-kidney), 93 transplants were conducted outside the program (with private or supplementary insurance as a funding source), and 5 patients were transplanted through the program but completed their follow-up outside the program. Among 944 patients, 217 grafts were lost (23.0%) until 2017: 102 (10.8%) due to death and 115 (12.2%) due to graft failure or primary non-function. The details concerning GL and death etiology were previously published (12). Therefore, the current cohort was composed of 115 recipients who survived the transplant and resumed dialysis due to graft failure (n=92) or primary non-function (n=23).
