# Supplementary material for: Long-term outcomes after kidney transplant failure and variables related to risk of death and probability of retransplant: Results from a single-center cohort study in Brazil
Source: PLoS One. 2021 Jan 20;16(1):e0245628. doi: 10.1371/journal.pone.0245628 (PMC7816974; doi:10.1371/journal.pone.0245628)
Supplement: S2 Appendix — (DOCX) [file pone.0245628.s002.docx]

**APPENDIX 2: Variables of interest, immunosuppression and prophylaxis approaches.**

The standard immunosuppression was a combination of a calcineurin inhibitor (cyclosporin or tacrolimus), mycophenolate, and prednisone unless the recipient had any contraindication for each compound, while the schedule was individualized. Patients who received grafts from deceased donors and highly sensitized recipients from living donors were induced with thymoglobulin unless any contraindication was identified. All of the patients received prophylaxis with trimethoprim-sulfamethoxazole against Pneumocystis jirovecii and albendazole or ivermectin against Strongyloides stercoralis. The strategy to reduce the risk of cytomegalovirus (CMV) disease was the preemptive treatment.
