# Supplementary material for: Long-term outcomes after kidney transplant failure and variables related to risk of death and probability of retransplant: Results from a single-center cohort study in Brazil
Source: PLoS One. 2021 Jan 20;16(1):e0245628. doi: 10.1371/journal.pone.0245628 (PMC7816974; doi:10.1371/journal.pone.0245628)
Supplement: S1 Table — (DOCX) [file pone.0245628.s003.docx]

**S1 Table. Median of mismatches in ABDR *loci* in the first and in the second transplant.**

| **Variables** | **1^st^ Transplant** | **2^nd^ Transplant** | **P** |
| --- | --- | --- | --- |
| MMA | 1.0 (1.0; 2.0) | 1.0 (0.0; 2.0) | 0.66 |
| MMB | 1.0 (1.0; 2.0) | 1.0 (1.0; 2.0) | 0.82 |
| MMDR | 1.0 (0.0; 1.0) | 1.0 (0.25; 2.0) | 0.14 |
| Sum of MM | 3.0 (2.0; 4.0) | 2.0 (3.0; 5.0) | 0.94 |

Legend:

MM: mismatch.
