# Supplementary material for: Long-term outcomes after kidney transplant failure and variables related to risk of death and probability of retransplant: Results from a single-center cohort study in Brazil
Source: PLoS One. 2021 Jan 20;16(1):e0245628. doi: 10.1371/journal.pone.0245628 (PMC7816974; doi:10.1371/journal.pone.0245628)
Supplement: Attachment — Submitted filename: Response to Reviewers.docx [file pone.0245628.s005.docx]

Dear Academic Editor

Justyna Gołębiewska

On behalf of authors, we thank the opportunity to resubmit to **PLOS ONE** the revised version of our manuscript entitled **“Long-Term Outcomes after Kidney Transplant Failure and Variables Related to Risk of Death and Probability of Re-Transplant: Results from a Single-Center Cohort Study in Brazil”** (PONE-D-20-25397).

Your comments as well as the comments of the reviewers were crucial to improve our manuscript and all issues raised by them are following addressed.

Lúcio Roberto Requião-Moura

**Review**

**Academic editor**

*This manuscript is of interest to the renal transplant community, but is not acceptable for publication in its current form. The manuscript requires more detailed methodology and results description. Please provide data on:*

| **From the Editor:** | **Author comments:** |
| --- | --- |
| *time to retransplantation in those who got a second kidney from a living donor vs deceased donor* | Time to retransplant trended to be shorter in those who received the subsequent kidney from a living donor: 21.0 (8.1; 34.3) vs. 39.2 (15.5; 55.0) months, P=0.07. This information was included in the result section, as well in the table 3A (footnotes). |
| *causes of death on the waiting list* | The main cause of death was infection, with 35.9% of deaths being due to sepsis with the primary site of infection being well defined and 15.4% due to multiple organ and system dysfunction (MODS) without a primary site of infection being well defined or documented.  The following causes were cardiovascular events (15.4%) and cancer (10.2%). The deaths due to cancer were based on 4 events: melanoma, adenocarcinoma of the lung, carcinoma of bladder and sarcoma of unknown primary site.  In 12.7% of deaths it was not possible to establish their causes, while cirrhosis, trauma, acute complications of diabetes and uremia occurred in one patient (2.6%) each one. This last was the cause of death in a highly sensitized patient (PRA 100%) with dialysis access failure.  These details were added in the description of the results. |
| *the patients who died were older and more likely to have diabetes – what was the general burden of comorbidities eg. Measured by Charlson Comorbidity Index in both groups? What was the prevalence of chronic heart failure? Other cardiovascular diseases in those who died vs those who survived?* | The general burden of comorbidities at the moment when patients had resumed to dialysis, measured by Charlson comorbidity index (CCI), was 3.0 (2.0; 5.0), mainly due to the presence of chronic kidney disease in all patients.  Moreover, the CCI was significantly higher among patients who died: 5.0 (3.0; 6.0) vs. 2.0 (2.0; 3.0), P<0.001. There were no differences in the frequency of previous acute myocardial infarction (P=0.10) or heart failure (0.63) between patients who died vs. those who did not. These results were included in the table 4, as well as it was described in the result section.  Considering these results, the Cox regression models had to be rebuilt to include CCI as an independent variable (as well as residual graft function). In the current model, the risk of death was greatly affected by CCI: HR for each point= 1.29; 95%CI=1.10-1.51; P=0.002 (table 5). |
| *What was the general burden of comorbidities eg. Measured by Charlson Comorbidity Index, what was the prevalence of chronic heart failure and other cardiovascular diseases in those who got a second kidney transplant vs those who did not?* | The Charlson comorbidity index (CCI) was significantly higher among patients who did not get a following transplant: 3.5 (2.0; 6.0) vs. 2.0 (2.0; 3.0), P<0.001. There were no differences in the frequency of previous acute myocardial infarction (P=0.12) or heart failure (0.89) according to retransplant status. These results were included in the table 1, as well as it was described in the result section.  The Cox regression models was rebuilt to include CCI as an independent variable (as well as residual graft function), however the final result was not affected by these two factors (table 2). |
| *Did the probability of retransplantation differ between those with primary non function vs late kidney allograft failure?* | The probability of retransplantation was not different between those with primary loss, in comparison with late graft failure. The frequency of primary loss was 25.6% (based on 11 events) among patients who were retransplanted, while it was 16.7% (based on 12 events) in those who were not; P=0.25. These data are showed in the table 1.  Categorizing the primary losses in thrombosis and primary nonfunction (PNF), there were no differences between the frequency of events according to retransplant status (Fisher’s exact test P=0.57).  Nineteen patients had their grafts lost due to thrombosis (8 of them who were retransplanted and 11 who were not) and 3 due to PNF (2 of them who were retransplanted and 1 who was not).  These details were included in the table 1 (footnotes). |
| *What was the residual kidney allograft function at the time of referral to dialysis in different groups?* | The residual kidney allograft function at the time of referral to dialysis was estimated by CKD-epi equation.  The median of estimated glomerular filtration ratio (eGFR) was 7.2 (5.5; 9.2) mL/min/1.73 m^2^, being higher among patients who did not give a following transplant: 8.3 (5.8; 10.3) vs. 6.8 (5.2; 7.6), P=0.01. Therefore, the residual function was included in the modeling for Cox regression for probability of retransplantation, however it did not significantly impact the final results (table 2).  Moreover, eGFR was significantly higher among patients who died after kidney failure: 8.8 (6.5; 11.5) vs. 6.8 (5.2; 8.3), P=0.001. This association has been previously described in other cohorts (Kidney Int. 2002;62(5):1875; Molnar et al. Nephrol Dial Transplant, 2012; 27: 2913).  In the same way, the residual function was included in the modeling for Cox regression for probability of death.  Each mL/min/1.73 m^2^ of eGFR before resuming dialysis increased the probability of death in 14% (table 5): HR=1.14; 95%=1.04-1.25; P=0.004. |
| *When were the laboratory parameters such as Pi, PTH, K, Hgb collected – at the time of referral to dialysis, after starting dialysis? Please clarify* | Levels of hemoglobin, urea and potassium were collected on the same day when the patient had resumed to dialysis, until 7 days before and 14 or more days before in 69%, 16.9% and 14.1% of patients, respectively.  Levels of calcium and phosphorous were collected on the same day when the patient had resumed to dialysis, until 7 days before and 14 or more days before in 54.6%, 20.2% and 25.2% patients, respectively.    The median time when the PTH level was obtained was 30 (0; 158) days before the day when the patients had resumed to dialysis; 35% of the levels were obtained on the same day when the patients had resumed to dialysis.  We have included these information in the appendix 2, where the variables are detailed. |
| *page 9, lines 218-220 – “In the Cox regression, the risk of death was significantly higher in (…) and in those with a previous transplant history (HR=2.05; 95% CI=1.03-4.10; P=0.04).” – but all patients included in the analysis had a previous transplant history? – please clarify* | Among all 115 patients included in this cohort, 9 of them had a previous engraftment, and It was more frequent among patients who died after the graft loss (P=0.09, in table 4).  In the current version, we have presented a rebuilt modeling for Cox regression, including two other variables that have been suggested to you and reviewers: CCI and residual eGFR. When the model was adjusted for these variables, graft nephrectomy was not an independently (and statistically significant) variable associated with the risk of death. |

**Reviewer #1**

*I agree with the authors about need for more regional and local data in failing/failed kidney allograft population. As authors pointed out, only limited conclusions can be drawn from this single-center data set in terms of factors affecting re-transplant and dialysis. Regardless, some of the results of this analysis are thought provoking and merit further investigation. The data set is also limited in terms of the risk factors that were assessed.*

| **Reviewer 1 comments:** | **Author comments:** |
| --- | --- |
| *Can the authors comment on possibility of including patients comorbidities, especially cardiac and malignancy related, in risk factors. Also will the authors be able to describe the immunosuppression that patients were on after failed allograft, and if that affected outcomes?* | Thank you for this very important suggestion. As the academic editor has suggested, we included the Charlson comorbidity index (CCI) at the time to resuming dialysis.  The CCI was significantly higher among patients who did not get a following transplant: 3.5 (2.0; 6.0) vs. 2.0 (2.0; 3.0), P<0.001 (table 1). However, in the Cox regression it was not associated with the probability of retransplantation (table 2).  Moreover, the CCI was significantly higher among patients who died: 5.0 (3.0; 6.0) vs. 2.0 (2.0; 3.0), P<0.001 (table 4). In a new modeling for Cox regression, including CCI (as well residual graft function), the risk of death was greatly affected by CCI: HR for each point= 1.29; 95%CI=1.10-1.51; P=0.002 (table 5).  The immunosuppression that patients were on after failed allograft was described in the methods. However, we have added some details to clarify it: “*In terms of immunosuppression, only low-dose prednisone (5 mg/day) was sustained for at least 6 months unless the patient underwent graft nephrectomy when immunosuppression was withdrawn. At the time when patients had resumed dialysis other immunosuppressive drugs different from prednisone were withdrawn*”  In the Cox regression, the risk of death was affected by prednisone sustained: HR=3.37; 95% CI 1.54-7.40; P=0.002 (table 5). |
| *Line 42: Does AV graft include AV graft and AV fistula? If so, please change the term* | We have changed the term graft to fistula. This term was changed in the whole manuscript too. |
| *Line 44-45 :“In a sensitivity analysis, other two variables were related to morality: cellular acute rejection (B=- 0.859; P=0.02) and phosphatemia when resuming dialysis”*  *-Spelling correction: MORTALITY*  *-Suggest changing the language to clarify the effect of phosphorus. perhaps change to hyperphosphatemia* | We are very sorry for the inadvertent mistake about spelling of the word “mortality”.  Thank you for your suggestion. We have changed phosphatemia to hyperphosphatemia. |
| *3)Line 241-242; “Generally, the outcomes after kidney transplant are closed when the recipient returns to dialysis”. Change to “monitoring of the outcomes”*  *4)Line 285: “confection” . change to “construction”*  *5)line 339: “anti-HLA development” change to anti-HLA antibody development.* | Thank you for your suggestion. We have changed all of them. |
| *6)Line 365-366” Moreover, because this was a retrospective study, some variables had many missing values, mainly during the critical patient lifetime period, which is the new incidence in dialysis.”. Need language change. Not sure what "which is the new incidence in dialysis" ,means?* | To clarify the sentence, we have changed “which is the new incidence in dialysis” to “***which is the time to resuming dialysis***”. |

**Reviewer #2**

*Requião-Moura et al analyzed the variables related to the re-transplant and death risk with data obtained from a single-center cohort. I have minor concerns about the manuscript in its current form.*

| **Reviewer 2 comments:** | **Author comments:** |
| --- | --- |
| *English should be fully reviewed* | A careful grammar and spelling revision was performed, including English proofreading by a company specialized in language editing. |
| *Abstract: Authors reported “B” values but not hazard ratios of mortality for acute rejection and hypophosphatemia: “In a sensitivity analysis, other two variables were related to morality: cellular acute rejection (B=-0.859; P=0.02) and phosphatemia when resuming dialysis (B=-0.350, P=0.007).” Besides, please correct “morality”* | Thank you for your comment.  The abstract was reviewed. |
| *- When were defined the variables related to resuming dialysis? A patient can re-start dialysis by haemodialysis the first two months and be transferred to peritoneal dialysis after that. Similarly, when was defined a patient as with a AV graft access? At the graft loss or one month later? Please, clarify. Similarly, when were obtained urea, potassium, calcium, P and PTH?* | Levels of hemoglobin, urea and potassium were collected on the same day when the patient had resumed to dialysis, until 7 days before and 14 or more days before in 69%, 16.9% and 14.1% of patients, respectively.  Levels of calcium and phosphorous were collected on the same day when the patient had resumed to dialysis, until 7 days before and 14 or more days before in 54.6%, 20.2% and 25.2% patients, respectively.    The median time when the PTH level was obtained was 30 (0; 158) days before the day when the patients had resumed to dialysis; 35% of the levels were obtained on the same day when the patients had resumed to dialysis.  We have included these information in the appendix 2, where the variables are detailed. |
| *- After graft loss, when and how was measured PRA? Sensitization is a well-defined risk factor for not receiving a re-transplant. Although there were many missing values that limit the analysis of PRA, it could be interesting to dichotomize the variable PRA the rule out its relationship with the retransplant risk.* | Before 2006, PRA was measured by CDC in our service. However, in the present cohort, only 3 patients had their PRA measured in that era, and the results were 0%, 0% and 88%.  In the others (83), it was calculated (cPRA) based on the Single Antigen methods. To estimate the panel of reactivity, the local Lab use the frequency of HLA observed in the pool of donors in the city of São Paulo. The cut off for anti HLA was 2,000 mfi. This information was included in the Appendix 2 (and in the table’s footnotes).  In 19 patients, the PRA was collected on the same day or before patients had their graft lost: 5.2 (0.0; 0.25) months. In 67, it was collected 12.9 (4.7; 29.6) months after to resuming dialysis.  As you have suggested, we dichotomized PRA using 50% as a cut off: we did not observed differences in the frequency of PRA > 50% in patients who did not retransplant, as well in those who died. We have included theses details in tables 1 and 4. As the number of missing values was high, this result should be considered carefully.  Unfortunately, it was not possible to include this variable in the multivariable models due to the great number of missing values. |
| *- Authors reported that graft nephrectomy was independently associated to a lower death risk after multivariate regression analysis (table 5). According to the provided results (page 10) graft nephrectomy was performed in 19 patients due to graft vascular thrombosis whereas in the others the transplant nephrectomy was carried out after graft loss. These two groups are quite different with respect to the inflammatory-associated status and I suggest analyzing the death risk excluding the group of graft nephrectomy due to early graft vascular thrombosis.* | Thank you for your suggestion.  The frequency of graft nephrectomy carried out after late graft loss was not different among patients who died (vs. those who did not): 30.8% (based on 12 events) vs. 40.8% (based on 31 events), P=0.29.  Although it had not been achieved the statistical level to enter in the modeling for Cox regression, we rebuilt extra models to predict the risk of death, as you have suggested. These models were adjusted for other variables, such as residual eGFR and Charlson comorbidity index.  Actually, graft nephrectomy was not independently associated with the risk of death. |
| *- Higher phosphatemia did relate to a lower death risk (page 9). In page 12, authors suggested that higher phosphorus level can be a marker of better nutrition. In order to analyze this point, it could be interesting to include into the analysis the value of serum albumin at resuming dialysis if it is available. Related to this, some inflammatory markers such as CRP would be also interesting for analyzing.* | We totally agree with your comment. Unfortunately, we had a great number of missing values for serum albumin (61) and C-reactive protein (25). In spite of this lack of information, we have included this parameters in our results (table 1 and 4).  We could not notice differences in these biomarkers according to outcomes, however the high number of missing values cannot allow us to interpret these results. Thus, they should be considered carefully. |
| *- Besides, it would be expected to detect an improvement in the mortality results over the years that compensates for the potential increase in the age of the recipients. Would it be possible to incorporate the transplant seasons into the analysis (eg: <2009 vs.> 2009).* | It is a very interesting point. Thank you for your suggestion.  We categorized patients according to transplant era (before 2009 or later), as you have suggested. Seventy one patients had been transplanted before 2009, while 44 had been in 2009 or later.  Firstly, we analyzed the frequency of retransplant status according to transplant era, and it was exactly the same in both eras (38% < 2009 vs. 36.4% $\geq$ 2009, P=0.99). This result was included in the table 1.  Finally, we analyzed the frequency of death (or not) according to transplant era, and it was exactly the same in both eras too (35.2 vs. 31.8%, P=0.84). This analysis was included in the table 1. |
| *- Table 4. Thymoglobulin induction – % (n) is reported twice in the table and with different values* | We are sorry for this mistake. The table 4 was corrected. |
| *- Please, provide the causes of death if available.* | The main cause of death was infection, with 35.9% of deaths being due to sepsis with the primary site of infection being well defined and 15.4% due to multiple organ and system dysfunction (MODS) without a primary site of infection being well defined or documented.  The following causes were cardiovascular events (15.4%) and cancer (10.2%). The deaths due to cancer were based on 4 events: melanoma, adenocarcinoma of the lung, carcinoma of bladder and sarcoma of unknown primary site.  In 12.7% of deaths it was not possible to establish their causes, while cirrhosis, trauma, acute complications of diabetes and uremia occurred in one patient (2.6%) each one. This last was the cause of death in a highly sensitized patient (PRA 100%) with dialysis access failure.  These details were added in the description of the results. |
