# Supplementary material for: Long-term outcomes after kidney transplant failure and variables related to risk of death and probability of retransplant: Results from a single-center cohort study in Brazil
Source: PLoS One. 2021 Jan 20;16(1):e0245628. doi: 10.1371/journal.pone.0245628 (PMC7816974; doi:10.1371/journal.pone.0245628)
Supplement: Attachment — Submitted filename: Response to Reviewers.docx [file pone.0245628.s006.docx]

Dear Academic Editor

Justyna Gołębiewska

On behalf of authors, I would like to thank you for the opportunity to resubmit agin to **PLOS ONE** the revised version of our manuscript entitled **“Long-Term Outcomes after Kidney Transplant Failure and Variables Related to Risk of Death and Probability of Re-Transplant: Results from a Single-Center Cohort Study in Brazil”** (PONE-D-20-25397).

Your new comments were crucial to improve our manuscript and all issues raised by you are following addressed.

With my best regards,

Lúcio Roberto Requião-Moura

Rebuttal letter

| From the Editor | Answer from the authors |
| --- | --- |
| Please provide a more detailed methodology description - what kind of data was collected, at what time points? | In the previous version, the kind of data which was collected and their time points were detailed in the Appendix 2.  In the current version, we have moved these contents to the Methodology section. |
| Charlson comorbidity index should be described in the Methods section and requires citation | The Charlson comorbidity index was described in the Methods section and the citation was included in the current version. |
| When were the laboratory parameters such as Pi, PTH, K, Hgb collected – at the time of referral to dialysis, after starting dialysis? Please include this information in the methods section | In the previous version, this information was detailed in the Appendix 2. In the current one, we have moved these contents to the Methodology section.  These parameters were collected on the same day when patients had resumed to dialysis or before:  “*Levels of hemoglobin, urea and potassium were collected on the same day when the patient had resumed to dialysis, until 7 days before and 14 or more days before in 69%, 16.9% and 14.1% of patients, respectively. Levels of calcium and phosphorous were collected on the same day when the patient had resumed to dialysis, until 7 days before and 14 or more days before in 54.6%, 20.2% and 25.2% patients, respectively. The median time when the PTH level was obtained was 30 (0; 158) days before the day when the patients had resumed to dialysis; 35% of the levels were obtained on the same day when the patients had resumed to dialysis*”. |
| “In terms of immunosuppression, only low-dose prednisone (5 mg/day) was sustained for at least 6 months unless the patient underwent graft nephrectomy, when immunosuppression was withdrawn. At the time when patients had resumed dialysis other immunosuppressive drugs different from prednisone were withdrawn.”. At the same time “Finally, prednisone sustained after GL than doubled the risk of death: HR= 2.58; 95% CI= 258 1.24-5.35; P= 0.01”, but “The graft was removed from 62 patients (53.9%) and graft removal was more frequent among those who survived after GL: 60.5 vs 41.0%, P=0.05 (Table 4).” – Prednisone doubled the risk of death. It seems that prednisone was sustained if the graft was not removed and those who survived were more likely to have the graft removed. So was it the prednisone that doubled the risk of death? Or perhaps just older patients with more comorbidities were less likely to be referred for allograft nephrectomy, and for this reason were maintained on prednisone? Or perhaps had an underlying condition that required prednisone administration such as vasculitis, that overall increased the risk of death? – please clarify | Thank you for this very important comment. Actually, prednisone had been sustained for 6 months after graft loss in those whose renal graft was not removed (between graft loss and the first 6 months).  Moreover, the age at the time to resuming dialysis between patients who had the graft removed was not different from those who did not have (41.8 vs. 48.4 years old, p=0.76). We did not identify any underlying condition that required prednisone administration (such as vasculitis or any other).  Thus, prednisone sustained after graft loss was a colinear variable (with an inverse effect) with graft removal (including those that were performed due to thrombosis).  That is the reason why we had to rebuild the modeling for Cox regression, excluding this variable (prednisone sustained) from the model and keeping the graft removal due to thrombosis (as the reviewer #2 had suggested in the first round).  At this time the results were slightly different from the previous modeling. Furthermore, we have presented the current results in the table 5. Only two variables were independently associated with the risk of death: residual eGFR (HR=1.14; P=0.002) and Charlson comorbidity index (HR=1.37; P<0.001). Number of previous engraftment (P=0.05) and restarting dialysis by AV fistula (P=0.06) were run until the last step (7^th^ backward), but they did not reach a significant result. Acute rejection was automatically removed in the 7^th^ backward step. |
| At the same time “Having considered that are quite different with respect to the inflammatory-associated status between patients who underwent graft nephrectomy due to early thrombosis and those who underwent graft nephrectomy for other reasons, we analyzed the frequency of late nephrectomy among patients who died and those who did not. This frequency was lower among patients who died; however, this difference was not significant: 30.8% vs. 40.8%, P=0.29.”, but in the discussion “In our cohort, graft removal reduced the risk of mortality by 74%, but it was performed due to clinical indications in all of the patients.”– please clarify. | We are very sorry for this misinformation. The sentence in the Discussion was from the first version and it was maintained in the previous version inadvertently.  In the current version the sentence “*In our cohort, graft removal reduced the risk of mortality by 74%, but it was performed due to clinical indications in all of the patients*” was excluded. |
| What were the indications for late graft nephrectomy? | All of late graft nephrectomy were carried out due to pain, hematuria or, less commonly due to fever.  This content was included in the Discussion section. |
| “however, the frequency of patients who started hemodialysis by AV fistula graft access compared to hemodialysis using venous catheters or peritoneal dialysis was lower in the patients who died (51.3 vs 72.4%, P=0.02).” – started dialysis before the first renal transplantation or after graft loss? please clarify | Thank you for this comment. In fact, here we are talking about the time when patients had resumed to dialysis after graft loss.  To clarify it, this information was included in the sentence. |
| “this risk was associated with the mismatch in the HLA locus B.” – please discuss the possible explanation for this phenomenon. | When we removed the prednisone sustained as an independent variable from de Cox regression in the sensitivity analysis (to included level of phosphorous) the final result was the same those that we had observed in the first modeling. This is the reason why we have decided to exclude the supplementary table 5 in the current version.  At this time, the independent variable “level of phosphorous” was automatically removed from the Cox regression in the 4^th^ step, while the mismatch in the HLA locus B was in the 5^rd^ step. |
| “The median of the residual function assessed by estimated GFR was 7.2 mL/min/1.73 m2, and it was slightly higher (but statistically significant) among patients who died after GL (8.8 vs. 6.8, P=0.001). Each 1 mL/min/1.73 m2 increase in eGFR the risk of death by 14%. Although this result seems to be controversial, it has been observed in other cohorts.” – please discuss the possible explanation for this phenomenon. | In the current version we have discussed possible explanation for the relation between higher eGFR when patients resumed to dialysis and risk of death.  “*The median of the residual function assessed by estimated GFR was 7.2 mL/min/1.73 m^2^, and it was slightly higher (but statistically significant) among patients who died after GL (8.8 vs. 6.8, P=0.001). Each 1 mL/min/1.73 m^2^ increase in eGFR the risk of death by 14%. Although this result seems to be controversial, it has been observed in other cohorts. Earlier return to dialysis, defined as resuming dialysis when eGFR is higher than 10 mL/min/1.73 m^2^, seems to be associated with higher risk of mortality. Using a propensity score to evaluate the risk of death after transplant failure, Molnar et al demonstrated that the earlier return to dialysis was associated with diabetes mellitus (OR= 1.75) and peripheral vascular disease (OR= 3.55), two clinical conditions traditionally related with higher mortality (33). Having examined more than 4,700 patients from the United States Renal Data System (USRD) whose transplants failed, Gill et al observed that each 1 mL/min/1.73 m^2^ higher eGFR at dialysis reinitiation increased the risk of death by 4% (25). In the same way, in another large American cohort, Brar et al showed that eGFR < 10 mL/min/1.73 m^2^ at time of resuming dialysis reduced the risk of death by 17%. According to them, higher GFR alone would not be a good predictor of outcomes in patients who had resumed to dialysis, however it could be a marker of patients who are more unwell and they would have to be transitioned more quickly, mainly due to the presence of congestive heart failure, ischemic heart disease and cerebrovascular disease (34).”* |
