# Supplementary material for: Long-term outcomes after kidney transplant failure and variables related to risk of death and probability of retransplant: Results from a single-center cohort study in Brazil
Source: PLoS One. 2021 Jan 20;16(1):e0245628. doi: 10.1371/journal.pone.0245628 (PMC7816974; doi:10.1371/journal.pone.0245628)
Supplement: Attachment — Submitted filename: Response to Reviewers.docx [file pone.0245628.s007.docx]

Dear Academic Editor

Justyna Gołębiewska

On behalf of authors, I would like to thank you for the opportunity to resubmit to **PLOS ONE** the revised version of our manuscript entitled **“Long-Term Outcomes after Kidney Transplant Failure and Variables Related to Risk of Death and Probability of Re-Transplant: Results from a Single-Center Cohort Study in Brazil”** (PONE-D-20-25397).

You can find below our answer to the last request from the Academic Editor.

With my best regards,

Lúcio Roberto Requião-Moura

**Rebuttal letter:**

**From the Academic Editor**

In the Methods section the Authors state that "To comparison, the era of transplantation was  dichotomized in two periods: before 2009 and 2009 or later." However there are no results of such a comparison presented. Please clarify.

**From the authors:**

Thank you for your suggestion.

In the previous version the frequency of retransplant according to the era of transplantation (before 2009 or later) was described in the Table 1. As you have requested, in the current version, we are embodying these results in the subsection “*Characteristics associated with retransplantation*”, as following:

“*The frequency of retransplant status according to transplant era, dichotomized in before 2009 or later, was not different in both eras: 38% < 2009 vs. 36.4%* $\geq$ *2009, P=0.99*”.

In the same way, in the previous version the frequency of death according to transplant era was described in the Table 4. As you have requested, in the current version, we are writing these results in the subsection “*Characteristics associated with the risk of death*” as described below:

“*Here, we analyzed the death rate according to transplant era (before 2009 or later) and it was the same in both eras (35.2 vs. 31.8%, P=0.84)*”.
