# Supplementary material for: Long-term outcomes after kidney transplant failure and variables related to risk of death and probability of retransplant: Results from a single-center cohort study in Brazil
Source: PLoS One. 2021 Jan 20;16(1):e0245628. doi: 10.1371/journal.pone.0245628 (PMC7816974; doi:10.1371/journal.pone.0245628)
Supplement: Peer review history

# Peer review: Decision Letter 0

**DOI:** 10.1371/journal.pone.0245628.r001

**Authors:** Justyna Gołębiewska

5 Oct 2020

PONE-D-20-25397

Long-Term Outcomes after Kidney Transplant Failure and Variables Related to Risk of Death and Probability of Re-Transplant: Results from a Single-Center Cohort Study in Brazil

PLOS ONE

Dear Dr. Requião-Moura,

Thank you for submitting your manuscript to PLOS ONE. After careful consideration, we feel that it has merit but does not fully meet PLOS ONE’s publication criteria as it currently stands. Therefore, we invite you to submit a revised version of the manuscript that addresses the points raised during the review process.

==============================

ACADEMIC EDITOR:

This manuscript is of interest to the renal transplant community, but is not acceptable for publication in its current form. The manuscript requires more detailed methodology and results description.

Please provide data on:

- time to retransplantation in those who got a second kidney from a living donor vs deceased donor
- causes of death on the waiting list
- the patients who died were older and more likely to have diabetes – what was the general burden of comorbidities eg. measured by Charlson Comorbidity Index in both groups? what was the prevalence of chronic heart failure? other cardiovascular diseases in those who died vs those who survived?
- what was the general burden of comorbidities eg. measured by Charlson Comorbidity Index, what was the prevalence of chronic heart failure and other cardiovascular diseases in those who got a second kidney transplant vs those who did not?
- did the probability of retransplantation differ between those with primary non function vs late kidney allograft failure?
- what was the residual kidney allograft function at the time of referral to dialysis in different groups?
- when were the laboratory parameters such as Pi, PTH, K, Hgb collected – at the time of referral to dialysis, after starting dialysis? please clarify
- page 9, lines 218-220 – “In the Cox regression, the risk of death was significantly higher in (…) and in those with a previous transplant history (HR=2.05; 95% CI=1.03-4.10; P=0.04).” – but all patients included in the analysis had a previous transplant history? – please clarify

There are also other issues, which are described in detail by the Reviewers.

==============================

Please submit your revised manuscript by Nov 19 2020 11:59PM. If you will need more time than this to complete your revisions, please reply to this message or contact the journal office at plosone@plos.org. When you're ready to submit your revision, log on to https://www.editorialmanager.com/pone/ and select the 'Submissions Needing Revision' folder to locate your manuscript file.

Please include the following items when submitting your revised manuscript:

- A rebuttal letter that responds to each point raised by the academic editor and reviewer(s). You should upload this letter as a separate file labeled 'Response to Reviewers'.
- A marked-up copy of your manuscript that highlights changes made to the original version. You should upload this as a separate file labeled 'Revised Manuscript with Track Changes'.
- An unmarked version of your revised paper without tracked changes. You should upload this as a separate file labeled 'Manuscript'.

If you would like to make changes to your financial disclosure, please include your updated statement in your cover letter. Guidelines for resubmitting your figure files are available below the reviewer comments at the end of this letter.

If applicable, we recommend that you deposit your laboratory protocols in protocols.io to enhance the reproducibility of your results. Protocols.io assigns your protocol its own identifier (DOI) so that it can be cited independently in the future. For instructions see: http://journals.plos.org/plosone/s/submission-guidelines#loc-laboratory-protocols

We look forward to receiving your revised manuscript.

Kind regards,

Justyna Gołębiewska

Academic Editor

PLOS ONE

Journal requirements:

When submitting your revision, we need you to address these additional requirements.

1. Please ensure that your manuscript meets PLOS ONE's style requirements, including those for file naming. The PLOS ONE style templates can be found at

https://journals.plos.org/plosone/s/file?id=wjVg/PLOSOne_formatting_sample_main_body.pdf and

https://journals.plos.org/plosone/s/file?id=ba62/PLOSOne_formatting_sample_title_authors_affiliations.pdf

2. Thank you for stating that data were analysed anonymously. Please also clarify whether data was anonymized before researchers accessed the data, or whether researchers anonymized the data. If the need for consent was waived, please provide a waiver from your ethics committee as a supplementary file.

3. Please include your tables as part of your main manuscript and remove the individual files. Please note that supplementary tables (should remain/ be uploaded) as separate "supporting information" files

4.We note that you have indicated that data from this study are available upon request. PLOS only allows data to be available upon request if there are legal or ethical restrictions on sharing data publicly. For information on unacceptable data access restrictions, please see http://journals.plos.org/plosone/s/data-availability#loc-unacceptable-data-access-restrictions.

In your revised cover letter, please address the following prompts:

a) If there are ethical or legal restrictions on sharing a de-identified data set, please explain them in detail (e.g., data contain potentially identifying or sensitive patient information) and who has imposed them (e.g., an ethics committee). Please also provide contact information for a data access committee, ethics committee, or other institutional body to which data requests may be sent.

b) If there are no restrictions, please upload the minimal anonymized data set necessary to replicate your study findings as either Supporting Information files or to a stable, public repository and provide us with the relevant URLs, DOIs, or accession numbers. Please see http://www.bmj.com/content/340/bmj.c181.long for guidelines on how to de-identify and prepare clinical data for publication. For a list of acceptable repositories, please see http://journals.plos.org/plosone/s/data-availability#loc-recommended-repositories.

We will update your Data Availability statement on your behalf to reflect the information you provide.

5. Please include captions for your Supporting Information files at the end of your manuscript, and update any in-text citations to match accordingly. Please see our Supporting Information guidelines for more information: http://journals.plos.org/plosone/s/supporting-information.

[Note: HTML markup is below. Please do not edit.]

Reviewers' comments:

Reviewer's Responses to Questions

**Comments to the Author**

1. Is the manuscript technically sound, and do the data support the conclusions?

The manuscript must describe a technically sound piece of scientific research with data that supports the conclusions. Experiments must have been conducted rigorously, with appropriate controls, replication, and sample sizes. The conclusions must be drawn appropriately based on the data presented. 

Reviewer #1: Yes

Reviewer #2: Yes

**********

2. Has the statistical analysis been performed appropriately and rigorously? 

Reviewer #1: I Don't Know

Reviewer #2: Yes

**********

3. Have the authors made all data underlying the findings in their manuscript fully available?

The PLOS Data policy requires authors to make all data underlying the findings described in their manuscript fully available without restriction, with rare exception (please refer to the Data Availability Statement in the manuscript PDF file). The data should be provided as part of the manuscript or its supporting information, or deposited to a public repository. For example, in addition to summary statistics, the data points behind means, medians and variance measures should be available. If there are restrictions on publicly sharing data—e.g. participant privacy or use of data from a third party—those must be specified.

Reviewer #1: Yes

Reviewer #2: Yes

**********

4. Is the manuscript presented in an intelligible fashion and written in standard English?

PLOS ONE does not copyedit accepted manuscripts, so the language in submitted articles must be clear, correct, and unambiguous. Any typographical or grammatical errors should be corrected at revision, so please note any specific errors here.

Reviewer #1: Yes

Reviewer #2: Yes

**********

5. Review Comments to the Author

Please use the space provided to explain your answers to the questions above. You may also include additional comments for the author, including concerns about dual publication, research ethics, or publication ethics. (Please upload your review as an attachment if it exceeds 20,000 characters)

Reviewer #1: I agree with the authors about need for more regional and local data in failing/failed kidney allograft population. As authors pointed out, only limited conclusions can be drawn from this single-center data set in terms of factors affecting re-transplant and dialysis. Regardless, some of the results of this analysis are thought provoking and merit further investigation.

The data set is also limited in terms of the risk factors that were assessed. Can the authors comment on possibility of including patients comorbidities, especially cardiac and malignancy related, in risk factors. Also will the authors be able to describe the immunosuppression that patients were on after failed allograft, and if that affected outcomes?

I also have some minor suggestions:

1) Line 42: Does AV graft include AV graft and AV fistula? If so, please change the term

2) Line 44-45 :“In a sensitivity analysis, other two variables were related to morality: cellular acute rejection (B=- 0.859; P=0.02) and phosphatemia when resuming dialysis”

-Spelling correction: MORTALITY

-Suggest changing the language to clarify the effect of phosphorus. perhaps change to hyperphosphatemia

3)Line 241-242; “Generally, the outcomes after kidney transplant are closed when the recipient returns to dialysis”. Change to “monitoring of the outcomes”

4)Line 285: “confection” . change to “construction”

5)line 339: “anti-HLA development” change to anti-HLA antibody development

6)Line 365-366” Moreover, because this was a retrospective study, some variables had many missing values, mainly during the critical patient lifetime period, which is the new incidence in dialysis.”. Need language change. Not sure what "which is the new incidence in dialysis" ,means?

Reviewer #2: Requião-Moura et al analyzed the variables related to the re-transplant and death risk with data obtained from a single-center cohort. I have minor concerns about the manuscript in its current form.

- English should be fully reviewed

- Abstract: Authors reported “B” values but not hazard ratios of mortality for acute rejection and hypophosphatemia: “In a sensitivity analysis, other two variables were related to morality: cellular acute rejection (B=-0.859; P=0.02) and phosphatemia when resuming dialysis (B=-0.350, P=0.007).” Besides, please correct “morality”.

- When were defined the variables related to resuming dialysis? A patient can re-start dialysis by haemodialysis the first two months and be transferred to peritoneal dialysis after that. Similarly, when was defined a patient as with a AV graft access? At the graft loss or one month later? Please, clarify. Similarly, when were obtained urea, potassium, calcium, P and PTH?

- After graft loss, when and how was measured PRA? Sensitization is a well-defined risk factor for not receiving a re-transplant. Although there were many missing values that limit the analysis of PRA, it could be interesting to dichotomize the variable PRA the rule out its relationship with the retransplant risk.

- Authors reported that graft nephrectomy was independently associated to a lower death risk after multivariate regression analysis (table 5). According to the provided results (page 10) graft nephrectomy was performed in 19 patients due to graft vascular thrombosis whereas in the others the transplant nephrectomy was carried out after graft loss. These two groups are quite different with respect to the inflammatory-associated status and I suggest analyzing the death risk excluding the group of graft nephrectomy due to early graft vascular thrombosis.

- Higher phosphatemia did relate to a lower death risk (page 9). In page 12, authors suggested that higher phosphorus level can be a marker of better nutrition. In order to analyze this point, it could be interesting to include into the analysis the value of serum albumin at resuming dialysis if it is available. Related to this, some inflammatory markers such as CRP would be also interesting for analyzing.

- Besides, it would be expected to detect an improvement in the mortality results over the years that compensates for the potential increase in the age of the recipients. Would it be possible to incorporate the transplant seasons into the analysis (eg: <2009 vs.> 2009).

- Table 4. Thymoglobulin induction – % (n) is reported twice in the table and with different values

- Please, provide the causes of death if available.

**********

6. PLOS authors have the option to publish the peer review history of their article (what does this mean?). If published, this will include your full peer review and any attached files.

If you choose “no”, your identity will remain anonymous but your review may still be made public.

**Do you want your identity to be public for this peer review?** For information about this choice, including consent withdrawal, please see our Privacy Policy.

Reviewer #1: No

Reviewer #2: No

[NOTE: If reviewer comments were submitted as an attachment file, they will be attached to this email and accessible via the submission site. Please log into your account, locate the manuscript record, and check for the action link "View Attachments". If this link does not appear, there are no attachment files.]

While revising your submission, please upload your figure files to the Preflight Analysis and Conversion Engine (PACE) digital diagnostic tool, https://pacev2.apexcovantage.com/. PACE helps ensure that figures meet PLOS requirements. To use PACE, you must first register as a user. Registration is free. Then, login and navigate to the UPLOAD tab, where you will find detailed instructions on how to use the tool. If you encounter any issues or have any questions when using PACE, please email PLOS at figures@plos.org. Please note that Supporting Information files do not need this step.

# Author comment: Author response to Decision Letter 0

**DOI:** 10.1371/journal.pone.0245628.r002

3 Nov 2020

Dear Academic Editor

Justyna Gołębiewska

On behalf of authors, we thank the opportunity to resubmit to PLOS ONE the revised version of our manuscript entitled “Long-Term Outcomes after Kidney Transplant Failure and Variables Related to Risk of Death and Probability of Re-Transplant: Results from a Single-Center Cohort Study in Brazil” (PONE-D-20-25397).

Your comments as well as the comments of the reviewers were crucial to improve our manuscript and all issues raised by them are following addressed.

Lúcio Roberto Requião-Moura

Review 

Academic editor

This manuscript is of interest to the renal transplant community, but is not acceptable for publication in its current form. The manuscript requires more detailed methodology and results description. Please provide data on:

From the Editor:

time to retransplantation in those who got a second kidney from a living donor vs deceased donor 

Author comments:

Time to retransplant trended to be shorter in those who received the subsequent kidney from a living donor: 21.0 (8.1; 34.3) vs. 39.2 (15.5; 55.0) months, P=0.07. This information was included in the result section, as well in the table 3A (footnotes).

From the Editor:

causes of death on the waiting list 

Author comments:

The main cause of death was infection, with 35.9% of deaths being due to sepsis with the primary site of infection being well defined and 15.4% due to multiple organ and system dysfunction (MODS) without a primary site of infection being well defined or documented. 

The following causes were cardiovascular events (15.4%) and cancer (10.2%). The deaths due to cancer were based on 4 events: melanoma, adenocarcinoma of the lung, carcinoma of bladder and sarcoma of unknown primary site. 

In 12.7% of deaths it was not possible to establish their causes, while cirrhosis, trauma, acute complications of diabetes and uremia occurred in one patient (2.6%) each one. This last was the cause of death in a highly sensitized patient (PRA 100%) with dialysis access failure.

These details were added in the description of the results.

From the Editor:

the patients who died were older and more likely to have diabetes – what was the general burden of comorbidities eg. Measured by Charlson Comorbidity Index in both groups? What was the prevalence of chronic heart failure? Other cardiovascular diseases in those who died vs those who survived? 

Author comments:

The general burden of comorbidities at the moment when patients had resumed to dialysis, measured by Charlson comorbidity index (CCI), was 3.0 (2.0; 5.0), mainly due to the presence of chronic kidney disease in all patients.

Moreover, the CCI was significantly higher among patients who died: 5.0 (3.0; 6.0) vs. 2.0 (2.0; 3.0), P<0.001. There were no differences in the frequency of previous acute myocardial infarction (P=0.10) or heart failure (0.63) between patients who died vs. those who did not. These results were included in the table 4, as well as it was described in the result section. 

Considering these results, the Cox regression models had to be rebuilt to include CCI as an independent variable (as well as residual graft function). In the current model, the risk of death was greatly affected by CCI: HR for each point= 1.29; 95%CI=1.10-1.51; P=0.002 (table 5).

From the Editor:

What was the general burden of comorbidities eg. Measured by Charlson Comorbidity Index, what was the prevalence of chronic heart failure and other cardiovascular diseases in those who got a second kidney transplant vs those who did not? 

Author comments:

The Charlson comorbidity index (CCI) was significantly higher among patients who did not get a following transplant: 3.5 (2.0; 6.0) vs. 2.0 (2.0; 3.0), P<0.001. There were no differences in the frequency of previous acute myocardial infarction (P=0.12) or heart failure (0.89) according to retransplant status. These results were included in the table 1, as well as it was described in the result section.

The Cox regression models was rebuilt to include CCI as an independent variable (as well as residual graft function), however the final result was not affected by these two factors (table 2).

From the Editor:

Did the probability of retransplantation differ between those with primary non function vs late kidney allograft failure? 

Author comments:

The probability of retransplantation was not different between those with primary loss, in comparison with late graft failure. The frequency of primary loss was 25.6% (based on 11 events) among patients who were retransplanted, while it was 16.7% (based on 12 events) in those who were not; P=0.25. These data are showed in the table 1.

Categorizing the primary losses in thrombosis and primary nonfunction (PNF), there were no differences between the frequency of events according to retransplant status (Fisher’s exact test P=0.57). 

Nineteen patients had their grafts lost due to thrombosis (8 of them who were retransplanted and 11 who were not) and 3 due to PNF (2 of them who were retransplanted and 1 who was not).

These details were included in the table 1 (footnotes).

From the Editor:

What was the residual kidney allograft function at the time of referral to dialysis in different groups? 

Author comments:

The residual kidney allograft function at the time of referral to dialysis was estimated by CKD-epi equation.

The median of estimated glomerular filtration ratio (eGFR) was 7.2 (5.5; 9.2) mL/min/1.73 m2, being higher among patients who did not give a following transplant: 8.3 (5.8; 10.3) vs. 6.8 (5.2; 7.6), P=0.01. Therefore, the residual function was included in the modeling for Cox regression for probability of retransplantation, however it did not significantly impact the final results (table 2).

Moreover, eGFR was significantly higher among patients who died after kidney failure: 8.8 (6.5; 11.5) vs. 6.8 (5.2; 8.3), P=0.001. This association has been previously described in other cohorts (Kidney Int. 2002;62(5):1875; Molnar et al. Nephrol Dial Transplant, 2012; 27: 2913).

In the same way, the residual function was included in the modeling for Cox regression for probability of death. 

Each mL/min/1.73 m2 of eGFR before resuming dialysis increased the probability of death in 14% (table 5): HR=1.14; 95%=1.04-1.25; P=0.004.

From the Editor:

When were the laboratory parameters such as Pi, PTH, K, Hgb collected – at the time of referral to dialysis, after starting dialysis? Please clarify 

Author comments:

Levels of hemoglobin, urea and potassium were collected on the same day when the patient had resumed to dialysis, until 7 days before and 14 or more days before in 69%, 16.9% and 14.1% of patients, respectively.

Levels of calcium and phosphorous were collected on the same day when the patient had resumed to dialysis, until 7 days before and 14 or more days before in 54.6%, 20.2% and 25.2% patients, respectively.

The median time when the PTH level was obtained was 30 (0; 158) days before the day when the patients had resumed to dialysis; 35% of the levels were obtained on the same day when the patients had resumed to dialysis.

We have included these information in the appendix 2, where the variables are detailed.

From the Editor:

page 9, lines 218-220 – “In the Cox regression, the risk of death was significantly higher in (…) and in those with a previous transplant history (HR=2.05; 95% CI=1.03-4.10; P=0.04).” – but all patients included in the analysis had a previous transplant history? – please clarify 

Author comments:

Among all 115 patients included in this cohort, 9 of them had a previous engraftment, and It was more frequent among patients who died after the graft loss (P=0.09, in table 4).

In the current version, we have presented a rebuilt modeling for Cox regression, including two other variables that have been suggested to you and reviewers: CCI and residual eGFR. When the model was adjusted for these variables, graft nephrectomy was not an independently (and statistically significant) variable associated with the risk of death.

 

Reviewer #1

I agree with the authors about need for more regional and local data in failing/failed kidney allograft population. As authors pointed out, only limited conclusions can be drawn from this single-center data set in terms of factors affecting re-transplant and dialysis. Regardless, some of the results of this analysis are thought provoking and merit further investigation. The data set is also limited in terms of the risk factors that were assessed.

Reviewer 1 comments:

Can the authors comment on possibility of including patients comorbidities, especially cardiac and malignancy related, in risk factors. Also will the authors be able to describe the immunosuppression that patients were on after failed allograft, and if that affected outcomes? 

Author comments:

Thank you for this very important suggestion. As the academic editor has suggested, we included the Charlson comorbidity index (CCI) at the time to resuming dialysis.

The CCI was significantly higher among patients who did not get a following transplant: 3.5 (2.0; 6.0) vs. 2.0 (2.0; 3.0), P<0.001 (table 1). However, in the Cox regression it was not associated with the probability of retransplantation (table 2).

Moreover, the CCI was significantly higher among patients who died: 5.0 (3.0; 6.0) vs. 2.0 (2.0; 3.0), P<0.001 (table 4). In a new modeling for Cox regression, including CCI (as well residual graft function), the risk of death was greatly affected by CCI: HR for each point= 1.29; 95%CI=1.10-1.51; P=0.002 (table 5).

The immunosuppression that patients were on after failed allograft was described in the methods. However, we have added some details to clarify it: “In terms of immunosuppression, only low-dose prednisone (5 mg/day) was sustained for at least 6 months unless the patient underwent graft nephrectomy when immunosuppression was withdrawn. At the time when patients had resumed dialysis other immunosuppressive drugs different from prednisone were withdrawn”

In the Cox regression, the risk of death was affected by prednisone sustained: HR=3.37; 95% CI 1.54-7.40; P=0.002 (table 5).

Reviewer 1 comments:

Line 42: Does AV graft include AV graft and AV fistula? If so, please change the term 

Author comments:

We have changed the term graft to fistula. This term was changed in the whole manuscript too.

Reviewer 1 comments:

Line 44-45 :“In a sensitivity analysis, other two variables were related to morality: cellular acute rejection (B=- 0.859; P=0.02) and phosphatemia when resuming dialysis”

-Spelling correction: MORTALITY

-Suggest changing the language to clarify the effect of phosphorus. perhaps change to hyperphosphatemia 

Author comments:

We are very sorry for the inadvertent mistake about spelling of the word “mortality”.

Thank you for your suggestion. We have changed phosphatemia to hyperphosphatemia.

Reviewer 1 comments:

3)Line 241-242; “Generally, the outcomes after kidney transplant are closed when the recipient returns to dialysis”. Change to “monitoring of the outcomes”

4)Line 285: “confection” . change to “construction”

5)line 339: “anti-HLA development” change to anti-HLA antibody development. 

Author comments:

Thank you for your suggestion. We have changed all of them.

Reviewer 1 comments:

6)Line 365-366” Moreover, because this was a retrospective study, some variables had many missing values, mainly during the critical patient lifetime period, which is the new incidence in dialysis.”. Need language change. Not sure what "which is the new incidence in dialysis" ,means? 

Author comments:

To clarify the sentence, we have changed “which is the new incidence in dialysis” to “which is the time to resuming dialysis”.

 

Reviewer #2

Requião-Moura et al analyzed the variables related to the re-transplant and death risk with data obtained from a single-center cohort. I have minor concerns about the manuscript in its current form.

Reviewer 2 comments:

English should be fully reviewed 

Author comments:

A careful grammar and spelling revision was performed, including English proofreading by a company specialized in language editing.

Reviewer 2 comments:

Abstract: Authors reported “B” values but not hazard ratios of mortality for acute rejection and hypophosphatemia: “In a sensitivity analysis, other two variables were related to morality: cellular acute rejection (B=-0.859; P=0.02) and phosphatemia when resuming dialysis (B=-0.350, P=0.007).” Besides, please correct “morality” 

Author comments:

Thank you for your comment. 

The abstract was reviewed.

Reviewer 2 comments:

- When were defined the variables related to resuming dialysis? A patient can re-start dialysis by haemodialysis the first two months and be transferred to peritoneal dialysis after that. Similarly, when was defined a patient as with a AV graft access? At the graft loss or one month later? Please, clarify. Similarly, when were obtained urea, potassium, calcium, P and PTH? 

Author comments:

Levels of hemoglobin, urea and potassium were collected on the same day when the patient had resumed to dialysis, until 7 days before and 14 or more days before in 69%, 16.9% and 14.1% of patients, respectively.

Levels of calcium and phosphorous were collected on the same day when the patient had resumed to dialysis, until 7 days before and 14 or more days before in 54.6%, 20.2% and 25.2% patients, respectively.

The median time when the PTH level was obtained was 30 (0; 158) days before the day when the patients had resumed to dialysis; 35% of the levels were obtained on the same day when the patients had resumed to dialysis.

We have included these information in the appendix 2, where the variables are detailed.

Reviewer 2 comments:

- After graft loss, when and how was measured PRA? Sensitization is a well-defined risk factor for not receiving a re-transplant. Although there were many missing values that limit the analysis of PRA, it could be interesting to dichotomize the variable PRA the rule out its relationship with the retransplant risk.

Author comments:

Before 2006, PRA was measured by CDC in our service. However, in the present cohort, only 3 patients had their PRA measured in that era, and the results were 0%, 0% and 88%.

In the others (83), it was calculated (cPRA) based on the Single Antigen methods. To estimate the panel of reactivity, the local Lab use the frequency of HLA observed in the pool of donors in the city of São Paulo. The cut off for anti HLA was 2,000 mfi. This information was included in the Appendix 2 (and in the table’s footnotes).

In 19 patients, the PRA was collected on the same day or before patients had their graft lost: 5.2 (0.0; 0.25) months. In 67, it was collected 12.9 (4.7; 29.6) months after to resuming dialysis.

As you have suggested, we dichotomized PRA using 50% as a cut off: we did not observed differences in the frequency of PRA > 50% in patients who did not retransplant, as well in those who died. We have included theses details in tables 1 and 4. As the number of missing values was high, this result should be considered carefully.

Unfortunately, it was not possible to include this variable in the multivariable models due to the great number of missing values.

Reviewer 2 comments:

- Authors reported that graft nephrectomy was independently associated to a lower death risk after multivariate regression analysis (table 5). According to the provided results (page 10) graft nephrectomy was performed in 19 patients due to graft vascular thrombosis whereas in the others the transplant nephrectomy was carried out after graft loss. These two groups are quite different with respect to the inflammatory-associated status and I suggest analyzing the death risk excluding the group of graft nephrectomy due to early graft vascular thrombosis. 

Author comments:

Thank you for your suggestion. 

The frequency of graft nephrectomy carried out after late graft loss was not different among patients who died (vs. those who did not): 30.8% (based on 12 events) vs. 40.8% (based on 31 events), P=0.29. 

Although it had not been achieved the statistical level to enter in the modeling for Cox regression, we rebuilt extra models to predict the risk of death, as you have suggested. These models were adjusted for other variables, such as residual eGFR and Charlson comorbidity index. 

Actually, graft nephrectomy was not independently associated with the risk of death.

Reviewer 2 comments:

- Higher phosphatemia did relate to a lower death risk (page 9). In page 12, authors suggested that higher phosphorus level can be a marker of better nutrition. In order to analyze this point, it could be interesting to include into the analysis the value of serum albumin at resuming dialysis if it is available. Related to this, some inflammatory markers such as CRP would be also interesting for analyzing. 

Author comments:

We totally agree with your comment. Unfortunately, we had a great number of missing values for serum albumin (61) and C-reactive protein (25). In spite of this lack of information, we have included this parameters in our results (table 1 and 4).

We could not notice differences in these biomarkers according to outcomes, however the high number of missing values cannot allow us to interpret these results. Thus, they should be considered carefully.

Reviewer 2 comments:

- Besides, it would be expected to detect an improvement in the mortality results over the years that compensates for the potential increase in the age of the recipients. Would it be possible to incorporate the transplant seasons into the analysis (eg: <2009 vs.> 2009). 

Author comments:

It is a very interesting point. Thank you for your suggestion.

We categorized patients according to transplant era (before 2009 or later), as you have suggested. Seventy one patients had been transplanted before 2009, while 44 had been in 2009 or later.

Firstly, we analyzed the frequency of retransplant status according to transplant era, and it was exactly the same in both eras (38% < 2009 vs. 36.4% ≥ 2009, P=0.99). This result was included in the table 1.

Finally, we analyzed the frequency of death (or not) according to transplant era, and it was exactly the same in both eras too (35.2 vs. 31.8%, P=0.84). This analysis was included in the table 1.

Reviewer 2 comments:

- Table 4. Thymoglobulin induction – % (n) is reported twice in the table and with different values 

Author comments:

We are sorry for this mistake. The table 4 was corrected.

Reviewer 2 comments:

- Please, provide the causes of death if available. 

Author comments:

The main cause of death was infection, with 35.9% of deaths being due to sepsis with the primary site of infection being well defined and 15.4% due to multiple organ and system dysfunction (MODS) without a primary site of infection being well defined or documented. 

The following causes were cardiovascular events (15.4%) and cancer (10.2%). The deaths due to cancer were based on 4 events: melanoma, adenocarcinoma of the lung, carcinoma of bladder and sarcoma of unknown primary site. 

In 12.7% of deaths it was not possible to establish their causes, while cirrhosis, trauma, acute complications of diabetes and uremia occurred in one patient (2.6%) each one. This last was the cause of death in a highly sensitized patient (PRA 100%) with dialysis access failure.

These details were added in the description of the results.

Attachment Submitted filename: Response to Reviewers.docx Click here for additional data file.

# Peer review: Decision Letter 1

**DOI:** 10.1371/journal.pone.0245628.r003

**Authors:** Justyna Gołębiewska

23 Nov 2020

PONE-D-20-25397R1

Long-Term Outcomes after Kidney Transplant Failure and Variables Related to Risk of Death and Probability of Retransplant: Results from a Single-Center Cohort Study in Brazil

PLOS ONE

Dear Dr. Requião-Moura,

Thank you for submitting your manuscript to PLOS ONE. After careful consideration, we feel that it has merit but does not fully meet PLOS ONE’s publication criteria as it currently stands. Therefore, we invite you to submit a revised version of the manuscript that addresses the points raised during the review process.

==============================

ACADEMIC EDITOR:

Thank you for the changes introduced. However, the following issues still require clarification:

- Please provide a more detailed methodology description - what kind of data was collected, at what time points?
- Charlson comorbidity index should be described in the Methods section and requires citation
- When were the laboratory parameters such as Pi, PTH, K, Hgb collected – at the time of referral to dialysis, after starting dialysis? Please include this information in the methods section
- “In terms of immunosuppression, only low-dose prednisone (5 mg/day) was sustained for at least 6 months unless the patient underwent graft nephrectomy, when immunosuppression was withdrawn. At the time when patients had resumed dialysis other immunosuppressive drugs different from prednisone were withdrawn.”. At the same time “Finally, prednisone sustained after GL than doubled the risk of death: HR= 2.58; 95% CI= 258 1.24-5.35; P= 0.01”, but “The graft was removed from 62 patients (53.9%) and graft removal was more frequent among those who survived after GL: 60.5 vs 41.0%, P=0.05 (Table 4).” – Prednisone doubled the risk of death. It seems that prednisone was sustained if the graft was not removed and those who survived were more likely to have the graft removed. So was it the prednisone that doubled the risk of death? Or perhaps just older patients with more comorbidities were less likely to be referred for allograft nephrectomy, and for this reason were maintained on prednisone? Or perhaps had an underlying condition that required prednisone administration such as vasculitis, that overall increased the risk of death? – please clarify
- At the same time “Having considered that are quite different with respect to the inflammatory-associated status between patients who underwent graft nephrectomy due to early thrombosis and those who underwent graft nephrectomy for other reasons, we analyzed the frequency of late nephrectomy among patients who  died and those who did not. This frequency was lower among patients who died; however, this  difference was not significant: 30.8% vs. 40.8%, P=0.29.”, but in the discussion “In our cohort, graft removal reduced the risk of mortality by 74%, but it was performed due to clinical indications in all of the patients.”– please clarify.
- What were the indications for late graft nephrectomy?
- “however, the frequency of patients who started hemodialysis by AV fistula graft access compared to hemodialysis using venous catheters or peritoneal dialysis was lower in the patients who died (51.3 vs 72.4%, P=0.02).” – started dialysis before the first renal transplantation or after graft loss? please clarify
- “this risk was associated with the mismatch in the HLA locus B.” – please discuss the possible explanation for this phenomenon.
- “The median of the residual function assessed by estimated GFR was 7.2 mL/min/1.73 m2, and it was slightly higher (but statistically significant) among patients who died after GL (8.8 vs. 6.8, P=0.001).  Each 1 mL/min/1.73 m2 increase in eGFR the risk of death by 14%. Although this result seems to be  controversial, it has been observed in other cohorts.” – please discuss the possible explanation for this phenomenon

==============================

Please submit your revised manuscript by Jan 07 2021 11:59PM. If you will need more time than this to complete your revisions, please reply to this message or contact the journal office at plosone@plos.org. When you're ready to submit your revision, log on to https://www.editorialmanager.com/pone/ and select the 'Submissions Needing Revision' folder to locate your manuscript file.

Please include the following items when submitting your revised manuscript:

- A rebuttal letter that responds to each point raised by the academic editor and reviewer(s). You should upload this letter as a separate file labeled 'Response to Reviewers'.
- A marked-up copy of your manuscript that highlights changes made to the original version. You should upload this as a separate file labeled 'Revised Manuscript with Track Changes'.
- An unmarked version of your revised paper without tracked changes. You should upload this as a separate file labeled 'Manuscript'.

If you would like to make changes to your financial disclosure, please include your updated statement in your cover letter. Guidelines for resubmitting your figure files are available below the reviewer comments at the end of this letter.

If applicable, we recommend that you deposit your laboratory protocols in protocols.io to enhance the reproducibility of your results. Protocols.io assigns your protocol its own identifier (DOI) so that it can be cited independently in the future. For instructions see: http://journals.plos.org/plosone/s/submission-guidelines#loc-laboratory-protocols

We look forward to receiving your revised manuscript.

Kind regards,

Justyna Gołębiewska

Academic Editor

PLOS ONE

[Note: HTML markup is below. Please do not edit.]

Reviewers' comments:

Reviewer's Responses to Questions

**Comments to the Author**

1. If the authors have adequately addressed your comments raised in a previous round of review and you feel that this manuscript is now acceptable for publication, you may indicate that here to bypass the “Comments to the Author” section, enter your conflict of interest statement in the “Confidential to Editor” section, and submit your "Accept" recommendation.

Reviewer #1: All comments have been addressed

Reviewer #2: All comments have been addressed

**********

2. Is the manuscript technically sound, and do the data support the conclusions?

The manuscript must describe a technically sound piece of scientific research with data that supports the conclusions. Experiments must have been conducted rigorously, with appropriate controls, replication, and sample sizes. The conclusions must be drawn appropriately based on the data presented. 

Reviewer #1: Yes

Reviewer #2: Yes

**********

3. Has the statistical analysis been performed appropriately and rigorously? 

Reviewer #1: Yes

Reviewer #2: Yes

**********

4. Have the authors made all data underlying the findings in their manuscript fully available?

The PLOS Data policy requires authors to make all data underlying the findings described in their manuscript fully available without restriction, with rare exception (please refer to the Data Availability Statement in the manuscript PDF file). The data should be provided as part of the manuscript or its supporting information, or deposited to a public repository. For example, in addition to summary statistics, the data points behind means, medians and variance measures should be available. If there are restrictions on publicly sharing data—e.g. participant privacy or use of data from a third party—those must be specified.

Reviewer #1: Yes

Reviewer #2: Yes

**********

5. Is the manuscript presented in an intelligible fashion and written in standard English?

PLOS ONE does not copyedit accepted manuscripts, so the language in submitted articles must be clear, correct, and unambiguous. Any typographical or grammatical errors should be corrected at revision, so please note any specific errors here.

Reviewer #1: Yes

Reviewer #2: Yes

**********

6. Review Comments to the Author

Please use the space provided to explain your answers to the questions above. You may also include additional comments for the author, including concerns about dual publication, research ethics, or publication ethics. (Please upload your review as an attachment if it exceeds 20,000 characters)

Reviewer #1: (No Response)

Reviewer #2: The authors adequately addressed all questions and modified the manuscript accordingly. Although the risk of death and retransplantation after graft loss has been previously analyzed in other studies, there is no enough information on the long-tern outcome after graft loss in patients living in emerging and developing countries. The current manuscript provides this information.

**********

7. PLOS authors have the option to publish the peer review history of their article (what does this mean?). If published, this will include your full peer review and any attached files.

If you choose “no”, your identity will remain anonymous but your review may still be made public.

**Do you want your identity to be public for this peer review?** For information about this choice, including consent withdrawal, please see our Privacy Policy.

Reviewer #1: No

Reviewer #2: No

[NOTE: If reviewer comments were submitted as an attachment file, they will be attached to this email and accessible via the submission site. Please log into your account, locate the manuscript record, and check for the action link "View Attachments". If this link does not appear, there are no attachment files.]

While revising your submission, please upload your figure files to the Preflight Analysis and Conversion Engine (PACE) digital diagnostic tool, https://pacev2.apexcovantage.com/. PACE helps ensure that figures meet PLOS requirements. To use PACE, you must first register as a user. Registration is free. Then, login and navigate to the UPLOAD tab, where you will find detailed instructions on how to use the tool. If you encounter any issues or have any questions when using PACE, please email PLOS at figures@plos.org. Please note that Supporting Information files do not need this step.

# Author comment: Author response to Decision Letter 1

**DOI:** 10.1371/journal.pone.0245628.r004

14 Dec 2020

Dear Academic Editor

Justyna Gołębiewska

On behalf of authors, I would like to thank you for the opportunity to resubmit agin to PLOS ONE the revised version of our manuscript entitled “Long-Term Outcomes after Kidney Transplant Failure and Variables Related to Risk of Death and Probability of Re-Transplant: Results from a Single-Center Cohort Study in Brazil” (PONE-D-20-25397).

Your new comments were crucial to improve our manuscript and all issues raised by you are following addressed.

With my best regards,

Lúcio Roberto Requião-Moura

From the Editor:

Please provide a more detailed methodology description - what kind of data was collected, at what time points?

Answer from the authors:

In the previous version, the kind of data which was collected and their time points were detailed in the Appendix 2.

In the current version, we have moved these contents to the Methodology section.

From the Editor

Charlson comorbidity index should be described in the Methods section and requires citation

Answer from the authors

The Charlson comorbidity index was described in the Methods section and the citation was included in the current version.

From the Editor

When were the laboratory parameters such as Pi, PTH, K, Hgb collected – at the time of referral to dialysis, after starting dialysis? Please include this information in the methods section

Answer from the authors

In the previous version, this information was detailed in the Appendix 2. In the current one, we have moved these contents to the Methodology section.

These parameters were collected on the same day when patients had resumed to dialysis or before:

“Levels of hemoglobin, urea and potassium were collected on the same day when the patient had resumed to dialysis, until 7 days before and 14 or more days before in 69%, 16.9% and 14.1% of patients, respectively. Levels of calcium and phosphorous were collected on the same day when the patient had resumed to dialysis, until 7 days before and 14 or more days before in 54.6%, 20.2% and 25.2% patients, respectively. The median time when the PTH level was obtained was 30 (0; 158) days before the day when the patients had resumed to dialysis; 35% of the levels were obtained on the same day when the patients had resumed to dialysis”.

From the Editor

“In terms of immunosuppression, only low-dose prednisone (5 mg/day) was sustained for at least 6 months unless the patient underwent graft nephrectomy, when immunosuppression was withdrawn. At the time when patients had resumed dialysis other immunosuppressive drugs different from prednisone were withdrawn.”. At the same time “Finally, prednisone sustained after GL than doubled the risk of death: HR= 2.58; 95% CI= 258 1.24-5.35; P= 0.01”, but “The graft was removed from 62 patients (53.9%) and graft removal was more frequent among those who survived after GL: 60.5 vs 41.0%, P=0.05 (Table 4).” – Prednisone doubled the risk of death. It seems that prednisone was sustained if the graft was not removed and those who survived were more likely to have the graft removed. So was it the prednisone that doubled the risk of death? Or perhaps just older patients with more comorbidities were less likely to be referred for allograft nephrectomy, and for this reason were maintained on prednisone? Or perhaps had an underlying condition that required prednisone administration such as vasculitis, that overall increased the risk of death? – please clarify.

Answer from the authors

Thank you for this very important comment. Actually, prednisone had been sustained for 6 months after graft loss in those whose renal graft was not removed (between graft loss and the first 6 months). 

Moreover, the age at the time to resuming dialysis between patients who had the graft removed was not different from those who did not have (41.8 vs. 48.4 years old, p=0.76). We did not identify any underlying condition that required prednisone administration (such as vasculitis or any other). 

Thus, prednisone sustained after graft loss was a colinear variable (with an inverse effect) with graft removal (including those that were performed due to thrombosis). 

That is the reason why we had to rebuild the modeling for Cox regression, excluding this variable (prednisone sustained) from the model and keeping the graft removal due to thrombosis (as the reviewer #2 had suggested in the first round).

At this time the results were slightly different from the previous modeling. Furthermore, we have presented the current results in the table 5. Only two variables were independently associated with the risk of death: residual eGFR (HR=1.14; P=0.002) and Charlson comorbidity index (HR=1.37; P<0.001). Number of previous engraftment (P=0.05) and restarting dialysis by AV fistula (P=0.06) were run until the last step (7th backward), but they did not reach a significant result. Acute rejection was automatically removed in the 7th backward step.

From the Editor

At the same time “Having considered that are quite different with respect to the inflammatory-associated status between patients who underwent graft nephrectomy due to early thrombosis and those who underwent graft nephrectomy for other reasons, we analyzed the frequency of late nephrectomy among patients who died and those who did not. This frequency was lower among patients who died; however, this difference was not significant: 30.8% vs. 40.8%, P=0.29.”, but in the discussion “In our cohort, graft removal reduced the risk of mortality by 74%, but it was performed due to clinical indications in all of the patients.”– please clarify.

Answer from the authors

We are very sorry for this misinformation. The sentence in the Discussion was from the first version and it was maintained in the previous version inadvertently.

In the current version the sentence “In our cohort, graft removal reduced the risk of mortality by 74%, but it was performed due to clinical indications in all of the patients” was excluded. 

From the Editor

What were the indications for late graft nephrectomy?

Answer from the authors

All of late graft nephrectomy were carried out due to pain, hematuria or, less commonly due to fever.

This content was included in the Discussion section.

From the Editor

“however, the frequency of patients who started hemodialysis by AV fistula graft access compared to hemodialysis using venous catheters or peritoneal dialysis was lower in the patients who died (51.3 vs 72.4%, P=0.02).” – started dialysis before the first renal transplantation or after graft loss? please clarify

Answer from the authors

Thank you for this comment. In fact, here we are talking about the time when patients had resumed to dialysis after graft loss.

To clarify it, this information was included in the sentence.

From the Editor

“this risk was associated with the mismatch in the HLA locus B.” – please discuss the possible explanation for this phenomenon.

Answer from the authors

When we removed the prednisone sustained as an independent variable from de Cox regression in the sensitivity analysis (to included level of phosphorous) the final result was the same those that we had observed in the first modeling. This is the reason why we have decided to exclude the supplementary table 5 in the current version.

At this time, the independent variable “level of phosphorous” was automatically removed from the Cox regression in the 4th step, while the mismatch in the HLA locus B was in the 5rd step.

From the Editor

“The median of the residual function assessed by estimated GFR was 7.2 mL/min/1.73 m2, and it was slightly higher (but statistically significant) among patients who died after GL (8.8 vs. 6.8, P=0.001). Each 1 mL/min/1.73 m2 increase in eGFR the risk of death by 14%. Although this result seems to be controversial, it has been observed in other cohorts.” – please discuss the possible explanation for this phenomenon.

Answer from the authors

In the current version we have discussed possible explanation for the relation between higher eGFR when patients resumed to dialysis and risk of death.

“The median of the residual function assessed by estimated GFR was 7.2 mL/min/1.73 m2, and it was slightly higher (but statistically significant) among patients who died after GL (8.8 vs. 6.8, P=0.001). Each 1 mL/min/1.73 m2 increase in eGFR the risk of death by 14%. Although this result seems to be controversial, it has been observed in other cohorts. Earlier return to dialysis, defined as resuming dialysis when eGFR is higher than 10 mL/min/1.73 m2, seems to be associated with higher risk of mortality. Using a propensity score to evaluate the risk of death after transplant failure, Molnar et al demonstrated that the earlier return to dialysis was associated with diabetes mellitus (OR= 1.75) and peripheral vascular disease (OR= 3.55), two clinical conditions traditionally related with higher mortality (33). Having examined more than 4,700 patients from the United States Renal Data System (USRD) whose transplants failed, Gill et al observed that each 1 mL/min/1.73 m2 higher eGFR at dialysis reinitiation increased the risk of death by 4% (25). In the same way, in another large American cohort, Brar et al showed that eGFR < 10 mL/min/1.73 m2 at time of resuming dialysis reduced the risk of death by 17%. According to them, higher GFR alone would not be a good predictor of outcomes in patients who had resumed to dialysis, however it could be a marker of patients who are more unwell and they would have to be transitioned more quickly, mainly due to the presence of congestive heart failure, ischemic heart disease and cerebrovascular disease (34).”

Attachment Submitted filename: Response to Reviewers.docx Click here for additional data file.

# Editor report: Decision Letter 2

**DOI:** 10.1371/journal.pone.0245628.r005

**Authors:** Justyna Gołębiewska

23 Dec 2020

PONE-D-20-25397R2

Long-Term Outcomes after Kidney Transplant Failure and Variables Related to Risk of Death and Probability of Retransplant: Results from a Single-Center Cohort Study in Brazil

PLOS ONE

Dear Dr. Requião-Moura,

Thank you for submitting your manuscript to PLOS ONE. After careful consideration, we feel that it has merit but does not fully meet PLOS ONE’s publication criteria as it currently stands. Therefore, we invite you to submit a revised version of the manuscript that addresses the points raised during the review process.

==============================

ACADEMIC EDITOR:

In the Methods section the Authors state that "To comparison, the era of transplantation was  dichotomized in two periods: before 2009 and 2009 or later." However there are no results of such a comparison presented. Please clarify.Please make sure to read the text carefully and to correct all inconsistencies before sending another revised version.

==============================

Please submit your revised manuscript by Feb 06 2021 11:59PM. If you will need more time than this to complete your revisions, please reply to this message or contact the journal office at plosone@plos.org. When you're ready to submit your revision, log on to https://www.editorialmanager.com/pone/ and select the 'Submissions Needing Revision' folder to locate your manuscript file.

Please include the following items when submitting your revised manuscript:

- A rebuttal letter that responds to each point raised by the academic editor and reviewer(s). You should upload this letter as a separate file labeled 'Response to Reviewers'.
- A marked-up copy of your manuscript that highlights changes made to the original version. You should upload this as a separate file labeled 'Revised Manuscript with Track Changes'.
- An unmarked version of your revised paper without tracked changes. You should upload this as a separate file labeled 'Manuscript'.

If you would like to make changes to your financial disclosure, please include your updated statement in your cover letter. Guidelines for resubmitting your figure files are available below the reviewer comments at the end of this letter.

If applicable, we recommend that you deposit your laboratory protocols in protocols.io to enhance the reproducibility of your results. Protocols.io assigns your protocol its own identifier (DOI) so that it can be cited independently in the future. For instructions see: http://journals.plos.org/plosone/s/submission-guidelines#loc-laboratory-protocols

We look forward to receiving your revised manuscript.

Kind regards,

Justyna Gołębiewska

Academic Editor

PLOS ONE

[Note: HTML markup is below. Please do not edit.]

[NOTE: If reviewer comments were submitted as an attachment file, they will be attached to this email and accessible via the submission site. Please log into your account, locate the manuscript record, and check for the action link "View Attachments". If this link does not appear, there are no attachment files.]

While revising your submission, please upload your figure files to the Preflight Analysis and Conversion Engine (PACE) digital diagnostic tool, https://pacev2.apexcovantage.com/. PACE helps ensure that figures meet PLOS requirements. To use PACE, you must first register as a user. Registration is free. Then, login and navigate to the UPLOAD tab, where you will find detailed instructions on how to use the tool. If you encounter any issues or have any questions when using PACE, please email PLOS at figures@plos.org. Please note that Supporting Information files do not need this step.

# Author comment: Author response to Decision Letter 2

**DOI:** 10.1371/journal.pone.0245628.r006

25 Dec 2020

Dear Academic Editor

Justyna Gołębiewska

On behalf of authors, I would like to thank you for the opportunity to resubmit to PLOS ONE the revised version of our manuscript entitled “Long-Term Outcomes after Kidney Transplant Failure and Variables Related to Risk of Death and Probability of Re-Transplant: Results from a Single-Center Cohort Study in Brazil” (PONE-D-20-25397).

You can find below our answer to the last request from the Academic Editor.

With my best regards,

Lúcio Roberto Requião-Moura

Rebuttal letter:

From the Academic Editor

In the Methods section the Authors state that "To comparison, the era of transplantation was dichotomized in two periods: before 2009 and 2009 or later." However there are no results of such a comparison presented. Please clarify.

From the authors:

Thank you for your suggestion. 

In the previous version the frequency of retransplant according to the era of transplantation (before 2009 or later) was described in the Table 1. As you have requested, in the current version, we are embodying these results in the subsection “Characteristics associated with retransplantation”, as following:

“The frequency of retransplant status according to transplant era, dichotomized in before 2009 or later, was not different in both eras: 38% < 2009 vs. 36.4% ≥ 2009, P=0.99”.

In the same way, in the previous version the frequency of death according to transplant era was described in the Table 4. As you have requested, in the current version, we are writing these results in the subsection “Characteristics associated with the risk of death” as described below:

“Here, we analyzed the death rate according to transplant era (before 2009 or later) and it was the same in both eras (35.2 vs. 31.8%, P=0.84)”.

Attachment Submitted filename: Response to Reviewers.docx Click here for additional data file.

# Editor report: Decision Letter 3

**DOI:** 10.1371/journal.pone.0245628.r007

**Authors:** Justyna Gołębiewska

5 Jan 2021

Long-Term Outcomes after Kidney Transplant Failure and Variables Related to Risk of Death and Probability of Retransplant: Results from a Single-Center Cohort Study in Brazil

PONE-D-20-25397R3

Dear Dr. Requião-Moura,

We’re pleased to inform you that your manuscript has been judged scientifically suitable for publication and will be formally accepted for publication once it meets all outstanding technical requirements.

Within one week, you’ll receive an e-mail detailing the required amendments. When these have been addressed, you’ll receive a formal acceptance letter and your manuscript will be scheduled for publication.

An invoice for payment will follow shortly after the formal acceptance. To ensure an efficient process, please log into Editorial Manager at http://www.editorialmanager.com/pone/, click the 'Update My Information' link at the top of the page, and double check that your user information is up-to-date. If you have any billing related questions, please contact our Author Billing department directly at authorbilling@plos.org.

If your institution or institutions have a press office, please notify them about your upcoming paper to help maximize its impact. If they’ll be preparing press materials, please inform our press team as soon as possible -- no later than 48 hours after receiving the formal acceptance. Your manuscript will remain under strict press embargo until 2 pm Eastern Time on the date of publication. For more information, please contact onepress@plos.org.

Kind regards,

Justyna Gołębiewska

Academic Editor

PLOS ONE

Additional Editor Comments (optional):

Reviewers' comments:

# Editor report: Acceptance letter

**DOI:** 10.1371/journal.pone.0245628.r008

**Authors:** Justyna Gołębiewska

7 Jan 2021

PONE-D-20-25397R3 

Long-Term Outcomes after Kidney Transplant Failure and Variables Related to Risk of Death and Probability of Retransplant: Results from a Single-Center Cohort Study in Brazil 

Dear Dr. Requião-Moura:

I'm pleased to inform you that your manuscript has been deemed suitable for publication in PLOS ONE. Congratulations! Your manuscript is now with our production department. 

If your institution or institutions have a press office, please let them know about your upcoming paper now to help maximize its impact. If they'll be preparing press materials, please inform our press team within the next 48 hours. Your manuscript will remain under strict press embargo until 2 pm Eastern Time on the date of publication. For more information please contact onepress@plos.org.

If we can help with anything else, please email us at plosone@plos.org. 

Thank you for submitting your work to PLOS ONE and supporting open access. 

Kind regards, 

PLOS ONE Editorial Office Staff

on behalf of

Dr. Justyna Gołębiewska 

Academic Editor

PLOS ONE
